# Supplementary material for: A computational model for structural dynamics and reconfiguration of DNA assemblies
Source: Nat Commun. 2023 Nov 4;14:7079. doi: 10.1038/s41467-023-42873-4 (PMC10625641; doi:10.1038/s41467-023-42873-4)
Supplement: Supplementary file 1 — Supplementary Information [file 41467_2023_42873_MOESM1_ESM.pdf]

Supplementary Information

A computational model for structural dynamics and reconfiguration of DNA assemblies

Jae Young Lee[1], Heeyuen Koh[2], and Do-Nyun Kim[1,2,3,4*]

[1]Institute of Advanced Machines and Design, Seoul National University, 1 Gwanak-ro, Gwanak-gu, Seoul 08826, Korea
[2]Soft Foundry Institute, Seoul National University, 1 Gwanak-ro, Gwanak-gu, Seoul 08826, Korea
[3]Department of Mechanical Engineering, Seoul National University, 1 Gwanak-ro, Gwanak-gu, Seoul 08826, Korea
[4]Institute of Engineering Research, Seoul National University, 1 Gwanak-ro, Gwanak-gu, Seoul 08826, Korea
[*]Corresponding E-mail address: dnkim@snu.ac.kr

Table of contents

Supplementary Notes ... **4**

Supplementary Note 1. Langevin dynamics equation ... 4

1.1. Governing equations ... 4

Supplementary Note 2. Structural model ... 5

2.1. Structural finite element ... 5

2.2. Electrostatic finite element ... 6

2.3. Assembly ... 6

2.4. Mass matrix ... 6

Supplementary Note 3. Hydrodynamic model ... 7

3.1. Stokes flow ... 7

3.2. Generalized Rotne-Prager-Yamakawa matrix ... 7

Supplementary Note 4. Time integration ... 9

4.1. Derivation of the algorithm ... 9

4.2. Calculation of random force ... 12

4.3. Implementation ... 13

4.4. Linear analysis ... 14

Supplementary Note 5. Mode analysis ... 17

5.1. Principal component analysis ... 17

5.2. Normal mode analysis ... 17

Supplementary Note 6. Stacking model ... 18

6.1. Stacking energy ... 18

6.2. Finite element model ... 18

Supplementary Figures ... **20**

Supplementary Figure 1. Flow of the proposed dynamic framework ... 20

Supplementary Figure 2. Configurations of DNA wireframe structures with DX edges ... 21

Supplementary Figure 3. Configurations of DNA wireframe structures with 6HB edges ... 22

Supplementary Figure 4. RMSF distribution of DNA wireframe structures ... 23

Supplementary Figure 5. Comparison of DNA wireframe structures (3D) with cryo-EM data ... 24

Supplementary Figure 6. Comparison of DNA wireframe structures (2D) with cryo-EM data ... 25

Supplementary Figure 7. Dynamic simulations of DNA structures with modular dynamic units 26

Supplementary Figure 8. Dynamic simulations of reversible DNA structures ... 27

Supplementary Figure 9. Mode shapes of pointer structure using principal component analysis 28

Supplementary Figure 10. Mode shapes of pointer structure using normal mode analysis ... 29

Supplementary Figure 11. The ratio of broken base-pairs in the pointer structure ... 30

Supplementary Figure 12. Design of the 12HB structure ... 31

Supplementary Figure 13. Stacking distance trajectories in the switch structure 32
Supplementary Figure 14. Potential of mean force for stacking in the switch structure 33
Supplementary Figure 15. Trajectory of stacking energy in the switch structure 34
Supplementary Figure 16. The number of stacked base-pairs in the switch structure 35
Supplementary Figure 17. Local geometry of the switch structure at Mg^{2+} 25 mM 36
Supplementary Figure 18. Local geometry of the switch structure at Mg^{2+} 15 mM 37
Supplementary Figure 19. Local geometry of the switch structure at Mg^{2+} 10 mM 38
Supplementary Figure 20. Local geometry of the switch structure at Mg^{2+} 5 mM 39
Supplementary Figure 21. RMSF distribution of the switch structure at Mg^{2+} 25 mM 40
Supplementary Figure 22. RMSF distribution of the switch structure at Mg^{2+} 15 mM 41
Supplementary Figure 23. RMSF distribution of the switch structure at Mg^{2+} 10 mM 42
Supplementary Figure 24. RMSF distribution of the switch structure at Mg^{2+} 5 mM 43
Supplementary Figure 25. Correlation maps of the switch structure at Mg^{2+} 25 mM 44
Supplementary Figure 26. Correlation maps of the switch structure at Mg^{2+} 15 mM 45
Supplementary Figure 27. Correlation maps of the switch structure at Mg^{2+} 10 mM 46
Supplementary Figure 28. Correlation maps of the switch structure at Mg^{2+} 5 mM 47
Supplementary Figure 29. Stability region of a thermal harmonic oscillator. 48
Supplementary Figure 30. Numerical tests of a thermal harmonic oscillator 49
Supplementary Tables 50
Supplementary Table 1. RMSD of wireframe structures to static prediction for final 100 ns 50
Supplementary Table 2. Interior angle of wireframe structures 51
Supplementary Table 3. Out-of-plane angle of wireframe structures 52
Supplementary Table 4. Natural frequency of the pointer structure 53
Supplementary Table 5. System of MD simulations for the switch structure 54
Supplementary Table 6. Computing environment and performance for switch structure 55
Supplementary References 56

Supplementary Notes

Supplementary Note 1. Langevin dynamics equation

DNA structures are in an ionic solvent environment. However, considering all the degrees of freedom of the solvent molecules requires enormous computation and large memory. The Langevin dynamics equation provides a way to reduce the degrees of freedom to consider only the target system (DNA structure) in a heat bath. We assumed that the system mass of a DNA structure is large enough compared to the unit of surrounding solvent in the bath. The dynamic friction kernel was neglected, which implies that the heat bath has no memory effects on the system motion.

1.1. Governing equations

Accordingly, the dynamic behavior of the DNA structure can be expressed by the memoryless Langevin dynamics equation[1] for N degrees of freedom with the set of two matrix-form equations as

$$\dot{\mathbf{U}}^t = \mathbf{V}^t \tag{1.1}$$

$$\mathbf{M}\dot{\mathbf{V}}^t = \mathbf{F}^t - \mathbf{Z}^t\mathbf{V}^t + \mathbf{R}^t \tag{1.2}$$

where $\mathbf{U}^t$ and $\mathbf{V}^t$ are $6\mathrm{N} \times 1$ nodal coordinate and velocity vectors of the finite element assembly at time t, respectively, with $3\mathrm{N}$ translational and $3\mathrm{N}$ rotational degrees of freedom, $\mathbf{M}$ is a $6\mathrm{N} \times 6\mathrm{N}$ time-independent diagonal mass matrix, $\mathbf{F}^t$ is a $6\mathrm{N} \times 1$ internal force vector calculated from the configuration of structural finite elements as $\mathbf{F}^t = \mathbf{F}^t(\mathbf{U}^t)$, $\mathbf{Z}^t$ is a $6\mathrm{N} \times 6\mathrm{N}$ friction matrix, giving a mass-normalized one as $\boldsymbol{\gamma}^t = \mathbf{M}^{-1}\mathbf{Z}^t$, and $\mathbf{R}^t$ is a $6\mathrm{N} \times 1$ random force vector.

By the dissipation-fluctuation theorem, the random force was assumed to be Gaussian distributed with the statistical properties given by

$$\langle \mathbf{R}^t \rangle = 0 \tag{1.3}$$

$$\langle \mathbf{R}^t \mathbf{R}^\tau \rangle = 2\mathrm{k_B T}\mathbf{Z}^t\delta(t-\tau) \tag{1.4}$$

where $\mathrm{k_B}$ is the Boltzmann constant, and T is the absolute temperature of the heat bath.

To perform the Langevin dynamics simulation, we need to construct the internal force vector ($\mathbf{F}^t$), the mass matrix ($\mathbf{M}$), and the friction matrix ($\mathbf{Z}^t$) for time t. Then, the trajectory of a DNA structure is discretely updated by performing time integration: $[\mathbf{U}^t, \mathbf{V}^t] \rightarrow [\mathbf{U}^{t+\Delta t}, \mathbf{V}^{t+\Delta t}]$

Supplementary Note 2. Structural model

We describe the procedure for calculating the internal force vector and the mass matrix. For this, it is necessary to define the mechanical properties like the force-fields in molecular dynamics or coarse-grained models. We employed a multiscale framework for DNA structures, SNUPI[2], based on the finite element approach. Here, we briefly describe the general workflow of the structural model.

A base-pair was modeled as a node (or bead), and the physical interhelical or intrahelical connections in the DNA structure were expressed as structural finite elements. Electrostatic finite elements describe the distant electrostatic repulsion within the DNA structure. The resulting finite element assembly allows the calculation of internal force vectors for all degrees of freedom of the nodes.

2.1. Structural finite element

Specifically, a structural finite element connects two nodes with position and orientation information. Its coordinate vectors ($\mathbf{U}^{\mathrm{e}}$) and triad matrices ($\mathbf{T}^{\mathrm{e}}$) are given by

$$\mathbf{U}^{\mathrm{e}} = [\vec{\mathrm{u}}_1^{\mathrm{e}}, \vec{\mathrm{u}}_2^{\mathrm{e}}] \tag{2.1}$$

$$\mathbf{T}^{\mathrm{e}} = \left[\vec{\mathrm{t}}_1^{\mathrm{e}}, \vec{\mathrm{t}}_2^{\mathrm{e}}\right] \tag{2.2}$$

where $\vec{\mathrm{u}}_{\mathrm{n}}^{\mathrm{e}}$ and $\vec{\mathrm{t}}_{\mathrm{n}}^{\mathrm{e}}$ represent the 6×1 coordinate vector (translation and rotation) and 3×3 triad matrix of the nodes ($\mathrm{n} = 1,2$) in the element e, respectively.

The connecting type in a DNA structure can be categorized into intrahelical connection (base-pair steps), interhelical connection (crossover steps), and end-to-end connection of single-stranded DNA. Their unique properties were systematically characterized by molecular dynamics simulations[2-4]. Considering the connecting type and the composed sequences, a structural finite element has the information of six intrinsic geometric values ($\mathbf{G}^{\mathrm{e}}$), six mechanical rigidities ($\mathbf{R}^{\mathrm{e}}$), and fifteen coupling coefficients ($\mathbf{C}^{\mathrm{e}}$), depending on the sequence as follows.

$$\mathbf{G}^{\mathrm{e}} = \left[\Delta_{\mathrm{x}}, \Delta_{\mathrm{y}}, \Delta_{\mathrm{z}}, \Theta_{\mathrm{x}}, \Theta_{\mathrm{y}}, \Theta_{\mathrm{z}}\right]^{\mathrm{e}} \tag{2.3}$$

$$\mathbf{R}^{\mathrm{e}} = \left[\mathrm{EA}, \mathrm{GA}_{\mathrm{y}}, \mathrm{GA}_{\mathrm{z}}, \mathrm{GJ}, \mathrm{EI}_{\mathrm{y}}, \mathrm{EI}_{\mathrm{z}}\right]^{\mathrm{e}} \tag{2.4}$$

$$\mathbf{C}^{\mathrm{e}} = [\mathrm{g}(\Delta_{\mathrm{r}}, \Delta_{\mathrm{s}}), \mathrm{g}(\Theta_{\mathrm{r}}, \Theta_{\mathrm{s}}), \mathrm{g}(\Delta_{\mathrm{r}}, \Theta_{\mathrm{s}})]^{\mathrm{e}} \tag{2.5}$$

where Δ_i and Θ_i represent the relative geometric value for the translational and rotational degrees of freedom, respectively ($\mathrm{i} = \mathrm{x}, \mathrm{y}, \mathrm{z}$), the mechanical rigidities indicate the stretching (EA), shearing ($\mathrm{GA}_{\mathrm{y}}, \mathrm{GA}_{\mathrm{z}}$), torsional ($\mathrm{GJ}$), bending ($\mathrm{EI}_{\mathrm{y}}, \mathrm{EI}_{\mathrm{z}}$) terms, and the coupling coefficients are the three translational-translational ($\mathrm{g}(\Delta_{\mathrm{r}}, \Delta_{\mathrm{s}})$), three rotational-rotational ($\mathrm{g}(\Theta_{\mathrm{r}}, \Theta_{\mathrm{s}})$), and nine translational-rotational ($\mathrm{g}(\Delta_{\mathrm{r}}, \Theta_{\mathrm{s}})$) terms.

Using these variables in the local coordinate, the stiffness matrix and the internal force vector of the structural finite element (ST) can be expressed as follows.

$$\mathbf{K}_{\mathrm{ST,L}}^{\mathrm{e}} = \mathbf{K}_{\mathrm{ST,L}}^{\mathrm{e}}(\mathbf{G}^{\mathrm{e}}, \mathbf{R}^{\mathrm{e}}, \mathbf{C}^{\mathrm{e}}) \tag{2.6}$$

$$\mathbf{F}_{\mathrm{ST,L}}^{\mathrm{e}} = \mathbf{F}_{\mathrm{ST,L}}^{\mathrm{e}}(\mathbf{G}^{\mathrm{e}}, \mathbf{R}^{\mathrm{e}}, \mathbf{C}^{\mathrm{e}}) \tag{2.7}$$

where $\mathbf{K}_{\mathrm{ST,L}}^{\mathrm{e}}$ and $\mathbf{F}_{\mathrm{ST,L}}^{\mathrm{e}}$ are the local stiffness matrix and the internal force vector.

To convert the local variables into global ones, we introduced the co-rotational framework[5], whose idea is based on the decomposition of the rigid body motion and the deformation of the finite element in the local coordinate. Based on the coordinate information of the nodes (position and triad), the $6N \times 1$ internal force vector in the global coordinate ($\mathbf{F}^{e}_{ST,G}$) can be calculated as follows.

$$\mathbf{F}^{e}_{ST,G} = \mathbf{F}^{e}_{ST,G}(\mathbf{U}^{e}, \mathbf{T}^{e}, \mathbf{K}^{e}_{ST,L}(\mathbf{G}^{e}, \mathbf{R}^{e}, \mathbf{C}^{e}), \mathbf{F}^{e}_{ST,L}(\mathbf{G}^{e}, \mathbf{R}^{e}, \mathbf{C}^{e})) \qquad (2.8)$$

2.2. Electrostatic finite element

An electrostatic finite element connects two nodes with coordinate information and has electrostatic properties ($\mathbf{E}$) depending on the ionic solvent environment as follows.

$$\mathbf{E} = [q, \lambda_D(T, I)] \qquad (2.9)$$

where q represents the effective charge of each node, and λ_D is the Debye screening length as a function of absolute temperature T and ionic strength I.

From the variables, the $6N \times 1$ internal force vector of the electrostatic finite element in the global coordinate can be expressed as follows.

$$\mathbf{F}^{e}_{ES,G} = \mathbf{F}^{e}_{ES,G}(\mathbf{U}^{e}, \mathbf{E}) \qquad (2.10)$$

where $\mathbf{F}^{e}_{ES,G}$ has only translational terms and $\mathbf{U}^{e}$ is the coordinate vectors of the electrostatic finite element.

2.3. Assembly

Finally, we can assemble all structural and electrostatic finite elements to obtain the $6N \times 1$ internal force vectors ($\mathbf{F}_G$) given by

$$\mathbf{F}_G(\mathbf{U}, \mathbf{T}, \mathbf{G}, \mathbf{R}, \mathbf{C}, \mathbf{E}) = \text{assembly}\left(\mathbf{F}^{e}_{ST,G}(\mathbf{U}^{e}, \mathbf{T}^{e}, \mathbf{K}^{e}_{ST,L}(\mathbf{G}^{e}, \mathbf{R}^{e}, \mathbf{C}^{e}), \mathbf{F}^{e}_{ST,L}(\mathbf{G}^{e}, \mathbf{R}^{e}, \mathbf{C}^{e})) \cup \mathbf{F}^{e}_{ES,G}(\mathbf{U}^{e}, \mathbf{E})\right) \qquad (2.11)$$

This demonstrates that the internal force vector includes all the information on the coordinates ($\mathbf{U}$) and triads ($\mathbf{T}$) of the nodes, the structural properties ($\mathbf{G}, \mathbf{R}, \mathbf{C}$), and the electrostatic properties ($\mathbf{E}$).

2.4. Mass matrix

Assuming that each node (base-pair) is a cylinder with a short height, we can generate the $6N \times 6N$ mass matrix as

$$\mathbf{M} = \text{assembly}(\mathbf{m}^{e}) \qquad (2.12)$$

where $\mathbf{m}^{e}$ is the element mass matrix of structural finite elements given by

$$\mathbf{m}^{e} = \text{diag}([m \quad m \quad m \quad \alpha \quad \beta \quad \beta]) \qquad (2.13)$$

where the first three components are the translational degrees of freedom with the mass (m), and the rotational components were constructed using the cylindrical moments of inertia for torsion and bending as $\alpha = mL^2/12$ and $\beta = mR^2/2$, respectively. We used the sequence-dependent mass values of base-pairs from the previous report[6] as Adenine: 313.2096 Da, Guanine: 329.2089 Da, Cytosine: 289.1843 Da, and Thymine: 304.1959 Da.

Supplementary Note 3. Hydrodynamic model

The friction matrix can be derived by assuming the Stokes flow surrounding a DNA structure. In Stokes flow, the friction matrix can be obtained by calculating the inverse of the mobility matrix. Considering that we defined a node (base-pair) in the structural model to have 6 degrees of freedom (3 translations and 3 rotations), we employed the generalized Rotne-Prager-Yamakawa mobility[7] with the same degrees of freedom.

3.1. Stokes flow

Assuming that the external flow velocity, vorticity, and strain rate are zero, the linearity of the Stokes equations leads to the linear relationships between the flow parameters as

$$\mathbf{F} = \mathbf{Z}\mathbf{V} \tag{3.1}$$

or

$$\mathbf{V} = \boldsymbol{\Xi}\mathbf{F} \tag{3.2}$$

where the $\mathbf{Z}$ and $\boldsymbol{\Xi}$ are the $6\mathrm{N} \times 6\mathrm{N}$ friction and mobility matrices, and $\mathbf{F}$ and $\mathbf{V}$ are the $6\mathrm{N} \times 1$ friction force and velocity vectors, respectively as

$$\mathbf{F} = [\mathrm{F}_1 \quad \mathrm{W}_1 \quad \cdots \quad \mathrm{F}_\mathrm{N} \quad \mathrm{W}_\mathrm{N}]^\mathrm{T} \tag{3.3}$$

$$\mathbf{V} = [\mathrm{V}_1 \quad \Omega_1 \quad \cdots \quad \mathrm{V}_\mathrm{N} \quad \Omega_\mathrm{N}]^\mathrm{T} \tag{3.4}$$

Here, F_i and W_i represent translational and rotational friction forces and V_i and Ω_i represent translational and rotational velocities of the nodes, respectively. This relationship suggests that the friction matrix can be calculated by inverting the mobility matrix as $\mathbf{Z} = \boldsymbol{\Xi}^{-1}$. Since the direct calculation of the friction matrix is generally impossible, we obtained the friction matrix by calculating the mobility matrix and then inverting it.

3.2. Generalized Rotne-Prager-Yamakawa matrix

There are several approaches to constructing mobility matrices of a bead system, where each node is considered a spherical bead. Among them, we introduced the generalized Rotne-Prager-Yamakawa approximation[7-9], which provides the $6\mathrm{N} \times 6\mathrm{N}$ mobility matrix ($\boldsymbol{\Xi}$) from the coordinate vector of nodes ($\mathbf{U}$) for all translational and rotational degrees of freedom as finite elements in the structural model as below.

$$\boldsymbol{\Xi}(\mathbf{U}) = \mathrm{assembly}(\boldsymbol{\Xi}^\mathrm{e}(\mathbf{U}^\mathrm{e})) \tag{3.5}$$

where $\boldsymbol{\Xi}^\mathrm{e}$ is a 6×6 element mobility matrix, depending on the coordinate vector ($\mathbf{U}^\mathrm{e}$).

For the interaction between nodes m and n, the 6×6 element mobility matrix can be decomposed into 3×3 translational and rotational matrices by

$$\boldsymbol{\Xi}_\mathrm{mn} = \begin{bmatrix} \boldsymbol{\mu}_\mathrm{mn}^\mathrm{tt} & \boldsymbol{\mu}_\mathrm{mn}^\mathrm{tr} \\ \boldsymbol{\mu}_\mathrm{mn}^\mathrm{rt} & \boldsymbol{\mu}_\mathrm{mn}^\mathrm{rr} \end{bmatrix} \tag{3.6}$$

where $\boldsymbol{\mu}_\mathrm{mn}^\mathrm{tt}$ represents the translational mobility matrix, $\boldsymbol{\mu}_\mathrm{mn}^\mathrm{rr}$ represents the rotational mobility matrix, and $\boldsymbol{\mu}_\mathrm{mn}^\mathrm{rt}$ or $\boldsymbol{\mu}_\mathrm{mn}^\mathrm{tr}$ are the translational-rotational mobility matrix. We omitted the element symbol (e).

First, the translational mobility matrix ($\boldsymbol{\mu}_{mn}^{tt}$) is given as

$$\boldsymbol{\mu}_{mn}^{tt} = \begin{cases} \dfrac{1}{6\pi\eta\sigma}\mathbf{I} & (m = n) \\ \dfrac{1}{8\pi\eta r_{mn}}\left[\left(1 + \dfrac{2}{3}\dfrac{\sigma^2}{r_{mn}^2}\right)\mathbf{I} + \left(1 - 2\dfrac{\sigma^2}{r_{mn}^2}\right)\dfrac{\vec{r}_{mn}\vec{r}_{mn}^T}{r_{mn}^2}\right] & (m \neq n,\ \ r_{mn} > 2\sigma) \\ \dfrac{1}{6\pi\eta\sigma}\left[\left(1 - \dfrac{9}{32}\dfrac{r_{mn}}{\sigma}\right)\mathbf{I} + \dfrac{3}{32}\dfrac{\vec{r}_{mn}\vec{r}_{mn}^T}{\sigma r_{mn}^2}\right] & (m \neq n,\ \ r_{mn} \leq 2\sigma) \end{cases} \tag{3.7}$$

where we assumed the radius of the bead (node) to be $\sigma = 1.1$ nm and the dynamic viscosity of water at 25 °C as $\eta = 890$ μPa · s $(\mathrm{pN/nm^2 \cdot ps})$ from the previous studies[10,11], and $\mathbf{I}$ represents the 3×3 identity matrix, respectively, $\vec{r}_{mn}$ is the 3×1 coordinate vector between two node position vectors (u_i) as

$$\vec{r}_{mn} = \vec{u}_n - \vec{u}_m = [x_{mn} \quad y_{mn} \quad z_{mn}]^T \tag{3.8}$$

and r_{mn} is the magnitude of the coordinate vector. Next, the rotational mobility matrix ($\boldsymbol{\mu}_{mn}^{rr}$) is expressed as follows.

$$\boldsymbol{\mu}_{mn}^{rr} = \begin{cases} \dfrac{1}{8\pi\eta\sigma^3}\mathbf{I} & (m = n) \\ -\dfrac{1}{16\pi\eta r_{mn}^3}\left[I - 3\dfrac{\vec{r}_{mn}\vec{r}_{mn}^T}{r_{mn}^2}\right] & (m \neq n, r_{mn} > 2\sigma) \\ \dfrac{1}{8\pi\eta\sigma^3}\left[\left(1 - \dfrac{27}{32}\dfrac{r_{mn}}{\sigma} + \dfrac{5}{64}\dfrac{r_{mn}^3}{\sigma^3}\right)\mathbf{I} + \left(\dfrac{9}{32}\dfrac{r_{mn}}{\sigma} - \dfrac{3}{64}\dfrac{r_{mn}^3}{\sigma^3}\right)\dfrac{\vec{r}_{mn}\vec{r}_{mn}^T}{\sigma r_{mn}^2}\right] & (m \neq n, r_{mn} \leq 2\sigma) \end{cases} \tag{3.9}$$

Finally, the translational-rotational mobility matrix ($\boldsymbol{\mu}_{mn}^{rt}$ or $\boldsymbol{\mu}_{mn}^{tr}$) is described as follows.

$$\boldsymbol{\mu}_{mn}^{rt} = [\boldsymbol{\mu}_{mn}^{tr}]^T = \begin{cases} \mathbf{O} & (m = n) \\ -\dfrac{1}{8\pi\eta r_{mn}^3}\varepsilon \cdot \vec{r}_{mn} & (m \neq n,\ \ r_{mn} > 2\sigma) \\ -\dfrac{1}{16\pi\eta\sigma^2}\left(\dfrac{1}{\sigma} - \dfrac{3}{8}\dfrac{r_{mn}}{\sigma^2}\right)\varepsilon \cdot \vec{r}_{mn} & (m \neq n,\ \ r_{mn} \leq 2\sigma) \end{cases} \tag{3.10}$$

where $\mathbf{O}$ denotes a 3×3 zero matrix and the last term in each equation is given by

$$\varepsilon \cdot \vec{r}_{mn} = \begin{bmatrix} 0 & z_{mn} & -y_{mn} \\ -z_{mn} & 0 & x_{mn} \\ y_{mn} & -x_{mn} & 0 \end{bmatrix} \tag{3.11}$$

The $6N \times 6N$ friction matrix for all degrees of freedom is finally obtained by inverting the mobility matrix[12] as $\mathbf{Z}(\mathbf{U}) = \boldsymbol{\Xi}^{-1}(\mathbf{U})$.

Supplementary Note 4. Time integration

We constructed the internal force vector ($\mathbf{F}^t$), the mass matrix ($\mathbf{M}$), and the friction matrix ($\mathbf{Z}^t$) for time t. The temporal trajectory of a DNA structure can be discretely updated by performing time integration: $[\mathbf{U}^t, \mathbf{V}^t] \rightarrow [\mathbf{U}^{t+\Delta t}, \mathbf{V}^{t+\Delta t}]$. We developed an algorithm for time integration based on the Grønbech-Jensen Farago (GJF) scheme[13].

4.1. Derivation of the algorithm

We integrated Langevin equations over a time step (Δt) for $[t + \Delta t/2]$ and $[t + \Delta t]$ as

$$\int_t^{t+\Delta t} \dot{\mathbf{U}}^t \mathrm{dt} = \int_t^{t+\Delta t} \mathbf{V}^t \mathrm{dt} \tag{4.1}$$

$$\mathbf{M}\int_t^{t+\frac{\Delta t}{2}} \dot{\mathbf{V}}^t \mathrm{dt} = \int_t^{t+\Delta t/2} \mathbf{F}^t \mathrm{dt} - \mathbf{M}\int_t^{t+\frac{\Delta t}{2}} \boldsymbol{\gamma}^t \mathbf{V}^t \mathrm{dt} + \int_t^{t+\frac{\Delta t}{2}} \mathbf{R}^t \mathrm{dt} \tag{4.2}$$

$$\mathbf{M}\int_t^{t+\Delta t} \dot{\mathbf{V}}^t \mathrm{dt} = \int_t^{t+\Delta t} \mathbf{F}^t \mathrm{dt} - \mathbf{M}\int_t^{t+\Delta t} \boldsymbol{\gamma}^t \mathbf{V}^t \mathrm{dt} + \int_t^{t+\Delta t} \mathbf{R}^t \mathrm{dt} \tag{4.3}$$

where the mass-normalized friction matrix can be considered quasi-static and time-independent during this integration domain $[t, t + \Delta t]$, and it is updated over large time intervals, giving $\boldsymbol{\gamma}^t = \boldsymbol{\gamma}$.

The integrated random force vector was denoted for simplicity as below.

$$\boldsymbol{\beta}_t^{t+\frac{\Delta t}{2}} = \int_t^{t+\frac{\Delta t}{2}} \mathbf{R}^t \mathrm{dt} \tag{4.4}$$

$$\boldsymbol{\beta}_t^{t+\Delta t} = \int_t^{t+\Delta t} \mathbf{R}^t \mathrm{dt} = \int_t^{t+\frac{\Delta t}{2}} \mathbf{R}^t \mathrm{dt} + \int_{t+\frac{\Delta t}{2}}^{t} \mathbf{R}^t \mathrm{dt} = \boldsymbol{\beta}_t^{t+\frac{\Delta t}{2}} + \boldsymbol{\beta}_{t+\frac{\Delta t}{2}}^{t} \tag{4.5}$$

These are Gaussian random numbers with zero mean and variance, associated with friction matrices as

$$\langle \boldsymbol{\beta}_t^{t+\frac{\Delta t}{2}} \rangle = \langle \boldsymbol{\beta}_t^{t+\Delta t} \rangle = 0 \tag{4.6}$$

$$\langle \boldsymbol{\beta}_t^{t+\frac{\Delta t}{2}} \boldsymbol{\beta}_\tau^{\tau+\frac{\Delta t}{2}} \rangle = \mathrm{k_B T}\Delta t \mathbf{M}\boldsymbol{\gamma}\delta(t-\tau) \tag{4.7}$$

$$\langle \boldsymbol{\beta}_t^{t+\Delta t} \boldsymbol{\beta}_\tau^{\tau+\Delta t} \rangle = 2\mathrm{k_B T}\Delta t \mathbf{M}\boldsymbol{\gamma}\delta(t-\tau) \tag{4.8}$$

from which, two equations (4.2) and (4.3) were rewritten as

$$\mathbf{V}^{t+\frac{\Delta t}{2}} = \mathbf{V}^t + \mathbf{M}^{-1}\int_t^{t+\frac{\Delta t}{2}} \mathbf{F}^t \mathrm{dt} - \boldsymbol{\gamma}\left(\mathbf{U}^{t+\frac{\Delta t}{2}} - \mathbf{U}^t\right) + \mathbf{M}^{-1}\boldsymbol{\beta}_t^{t+\frac{\Delta t}{2}} \tag{4.9}$$

$$\mathbf{V}^{t+\Delta t} = \mathbf{V}^t + \mathbf{M}^{-1}\int_t^{t+\Delta t} \mathbf{F}^t \mathrm{dt} - \boldsymbol{\gamma}\left(\mathbf{U}^{t+\Delta t} - \mathbf{U}^t\right) + \mathbf{M}^{-1}\boldsymbol{\beta}_t^{t+\Delta t} \tag{4.10}$$

To integrate the equation (4.1), we employed the trapezoidal rule between velocity and coordinate with error of Δt^3 scale as

$$\mathbf{U}^{t+\frac{\Delta t}{2}} - \mathbf{U}^{t} = \int_{t}^{t+\frac{\Delta t}{2}} \mathbf{V}^{t}\mathrm{dt} \simeq \frac{\Delta t}{4}\left(\mathbf{V}^{t+\frac{\Delta t}{2}} + \mathbf{V}^{t}\right) \tag{4.11}$$

$$\mathbf{U}^{t+\Delta t} - \mathbf{U}^{t} = \int_{t}^{t+\Delta t} \mathbf{V}^{t}\mathrm{dt} \simeq \frac{\Delta t}{2}\left(\mathbf{V}^{t+\Delta t} + \mathbf{V}^{t}\right) \tag{4.12}$$

The velocity in the equation (4.9) was inserted into the equation (4.11), yielding the first coordinate relation for the time, $t + \frac{\Delta t}{2}$ as follows.

$$\mathbf{U}^{t+\frac{\Delta t}{2}} - \mathbf{U}^{t} = \frac{\Delta t}{4}\left(\mathbf{V}^{t+\frac{\Delta t}{2}} + \mathbf{V}^{t}\right) \tag{4.13}$$

$$\mathbf{U}^{t+\frac{\Delta t}{2}} - \mathbf{U}^{t} = \frac{\Delta t}{4}\left(\mathbf{V}^{t} + \mathbf{M}^{-1}\int_{t}^{t+\frac{\Delta t}{2}} \mathbf{F}^{t}\mathrm{dt} - \boldsymbol{\gamma}\left(\mathbf{U}^{t+\frac{\Delta t}{2}} - \mathbf{U}^{t}\right) + \mathbf{M}^{-1}\boldsymbol{\beta}_{t}^{t+\frac{\Delta t}{2}} + \mathbf{V}^{t}\right) \tag{4.14}$$

$$\mathbf{U}^{t+\frac{\Delta t}{2}} - \mathbf{U}^{t} + \frac{\Delta t}{4}\boldsymbol{\gamma}\left(\mathbf{U}^{t+\frac{\Delta t}{2}} - \mathbf{U}^{t}\right) = \frac{\Delta t}{2}\mathbf{V}^{t} + \frac{\Delta t}{4}\mathbf{M}^{-1}\int_{t}^{t+\frac{\Delta t}{2}} \mathbf{F}^{t}\mathrm{dt} + \frac{\Delta t}{4}\mathbf{M}^{-1}\boldsymbol{\beta}_{t}^{t+\frac{\Delta t}{2}} \tag{4.15}$$

$$\left(\mathbf{I} + \frac{\Delta t}{4}\boldsymbol{\gamma}\right)\left(\mathbf{U}^{t+\frac{\Delta t}{2}} - \mathbf{U}^{t}\right) = \Delta t\left(\frac{1}{2}\mathbf{V}^{t} + \frac{1}{4}\mathbf{M}^{-1}\int_{t}^{t+\frac{\Delta t}{2}} \mathbf{F}^{t}\mathrm{dt} + \frac{1}{4}\mathbf{M}^{-1}\boldsymbol{\beta}_{t}^{t+\frac{\Delta t}{2}}\right) \tag{4.16}$$

$$\mathbf{U}^{t+\frac{\Delta t}{2}} = \mathbf{U}^{t} + \Delta t\left(\mathbf{I} + \frac{\Delta t}{4}\boldsymbol{\gamma}\right)^{-1}\left(\frac{1}{2}\mathbf{V}^{t} + \frac{1}{4}\mathbf{M}^{-1}\int_{t}^{t+\frac{\Delta t}{2}} \mathbf{F}^{t}\mathrm{dt} + \frac{1}{4}\mathbf{M}^{-1}\boldsymbol{\beta}_{t}^{t+\frac{\Delta t}{2}}\right) \tag{4.17}$$

$$\mathbf{U}^{t+\frac{\Delta t}{2}} = \mathbf{U}^{t} + \Delta t\mathbf{j}\left(\frac{1}{2}\mathbf{V}^{t} + \frac{1}{4}\mathbf{M}^{-1}\int_{t}^{t+\frac{\Delta t}{2}} \mathbf{F}^{t}\mathrm{dt} + \frac{1}{4}\mathbf{M}^{-1}\boldsymbol{\beta}_{t}^{t+\frac{\Delta t}{2}}\right) \tag{4.18}$$

where

$$\mathbf{j} = \left(\mathbf{I} + \frac{\Delta t}{4}\boldsymbol{\gamma}\right)^{-1} \tag{4.19}$$

Similarly, by inserting the equation (4.10) into the equation (4.12), we can obtain the second coordinate relation for the time, $t + \Delta t$ as follows.

$$\mathbf{U}^{t+\Delta t} - \mathbf{U}^{t} = \frac{\Delta t}{2}\left(\mathbf{V}^{t+\Delta t} + \mathbf{V}^{t}\right) \tag{4.20}$$

$$\mathbf{U}^{t+\Delta t} - \mathbf{U}^{t} = \frac{\Delta t}{2}\left(\mathbf{V}^{t} + \mathbf{M}^{-1}\int_{t}^{t+\Delta t} \mathbf{F}^{t}\mathrm{dt} - \boldsymbol{\gamma}\left(\mathbf{U}^{t+\Delta t} - \mathbf{U}^{t}\right) + \mathbf{M}^{-1}\boldsymbol{\beta}_{t}^{t+\Delta t} + \mathbf{V}^{t}\right) \tag{4.21}$$

$$\mathbf{U}^{t+\Delta t} - \mathbf{U}^{t} + \frac{\Delta t}{2}\boldsymbol{\gamma}\left(\mathbf{U}^{t+\Delta t} - \mathbf{U}^{t}\right) = \Delta t\mathbf{V}^{t} + \frac{\Delta t}{2}\mathbf{M}^{-1}\int_{t}^{t+\Delta t} \mathbf{F}^{t}\mathrm{dt} + \frac{\Delta t}{2}\mathbf{M}^{-1}\boldsymbol{\beta}_{t}^{t+\Delta t} \tag{4.22}$$

$$\left(\mathbf{I} + \frac{\Delta t}{2}\boldsymbol{\gamma}\right)\left(\mathbf{U}^{t+\Delta t} - \mathbf{U}^{t}\right) = \Delta t\left(\mathbf{V}^{t} + \frac{1}{2}\mathbf{M}^{-1}\int_{t}^{t+\Delta t} \mathbf{F}^{t}\mathrm{dt} + \frac{1}{2}\mathbf{M}^{-1}\boldsymbol{\beta}_{t}^{t+\Delta t}\right) \tag{4.23}$$

$$\mathbf{U}^{t+\Delta t} = \mathbf{U}^t + \Delta t\left(\mathbf{I} + \frac{\Delta t}{2}\boldsymbol{\gamma}\right)^{-\mathbf{1}}\left(\mathbf{V}^t + \frac{1}{2}\mathbf{M}^{-1}\int_t^{t+\Delta t}\mathbf{F}^t \mathrm{dt} + \frac{1}{2}\mathbf{M}^{-1}\boldsymbol{\beta}_t^{t+\Delta t}\right) \tag{4.24}$$

$$\mathbf{U}^{t+\Delta t} = \mathbf{U}^t + \Delta t\mathbf{b}\left(\mathbf{V}^t + \frac{1}{2}\mathbf{M}^{-1}\int_t^{t+\Delta t}\mathbf{F}^t \mathrm{dt} + \frac{1}{2}\mathbf{M}^{-1}\boldsymbol{\beta}_t^{t+\Delta t}\right) \tag{4.25}$$

where

$$\mathbf{b} = \left(\mathbf{I} + \frac{\Delta t}{2}\boldsymbol{\gamma}\right)^{-1} \tag{4.26}$$

For the approximation of internal force vectors in the resulting equations (4.18) and (4.25), we used the trapezoidal and Simpson's rules as

$$\int_t^{t+\frac{\Delta t}{2}}\mathbf{F}^t \mathrm{dt} \simeq \frac{\Delta t}{2}\mathbf{F}^t \tag{4.27}$$

$$\int_t^{t+\Delta t}\mathbf{F}^t \mathrm{dt} \simeq \frac{\Delta t}{6}\left(\mathbf{F}^{t+\Delta t} + 4\mathbf{F}^{t+\frac{\Delta t}{2}} + \mathbf{F}^t\right) = \Delta t\mathbf{F}^{t+\frac{\Delta t}{2}} \tag{4.28}$$

where this relation was derived using linear interpolation as

$$\mathbf{F}^{t+\Delta t} = \frac{\mathbf{F}^{t+\frac{\Delta t}{2}} - \mathbf{F}^t}{t + \frac{\Delta t}{2} - t}(t + \Delta t - t) + \mathbf{F}^t = 2\mathbf{F}^{t+\frac{\Delta t}{2}} - \mathbf{F}^t \tag{4.29}$$

$$\frac{\Delta t}{6}\left(\mathbf{F}^{t+\Delta t} + 4\mathbf{F}^{t+\frac{\Delta t}{2}} + \mathbf{F}^t\right) = \frac{\Delta t}{6}\left(2\mathbf{F}^{t+\frac{\Delta t}{2}} - \mathbf{F}^t + 4\mathbf{F}^{t+\frac{\Delta t}{2}} + \mathbf{F}^t\right) = \Delta t\mathbf{F}^{t+\frac{\Delta t}{2}} \tag{4.30}$$

Finally, we can summarize the updating relation for the coordinate and velocity as follows. To update the velocity, we can use the equation (4.10).

$$\mathbf{U}^{t+\frac{\Delta t}{2}} = \mathbf{U}^t + \Delta t\mathbf{j}\left(\frac{1}{2}\mathbf{V}^t + \frac{\Delta t}{8}\mathbf{f}^t + \frac{1}{4}\boldsymbol{\theta}_t^{t+\frac{\Delta t}{2}}\right) \tag{4.31}$$

$$\mathbf{U}^{t+\Delta t} = \mathbf{U}^t + \Delta t\mathbf{b}\left(\mathbf{V}^t + \frac{\Delta t}{2}\mathbf{f}^{t+\frac{\Delta t}{2}} + \frac{1}{2}\boldsymbol{\theta}_t^{t+\Delta t}\right) \tag{4.32}$$

$$\mathbf{V}^{t+\Delta t} = \mathbf{V}^t + \frac{\Delta t}{6}\left(\mathbf{f}^{t+\Delta t} + 4\mathbf{f}^{t+\frac{\Delta t}{2}} + \mathbf{f}^t\right) - \boldsymbol{\gamma}\left(\mathbf{U}^{t+\Delta t} - \mathbf{U}^t\right) + \boldsymbol{\theta}_t^{t+\Delta t} \tag{4.33}$$

or

$$\mathbf{V}^{t+\Delta t} = \mathbf{a}\mathbf{V}^t + \frac{\Delta t}{6}\left(\mathbf{f}^{t+\Delta t} + 2(\mathbf{a} + \mathbf{b})\mathbf{f}^{t+\frac{\Delta t}{2}} + \mathbf{f}^t\right) + \mathbf{b}\boldsymbol{\theta}_t^{t+\Delta t} \tag{4.34}$$

with

$$\mathbf{j} = \left(\mathbf{I} + \tfrac{\Delta t}{4}\boldsymbol{\gamma}\right)^{-1}, \quad \mathbf{b} = \left(\mathbf{I} + \tfrac{\Delta t}{2}\boldsymbol{\gamma}\right)^{-1}, \ \mathbf{a} = 2\mathbf{b} - \mathbf{I} = \left(\mathbf{I} - \tfrac{\Delta t}{2}\boldsymbol{\gamma}\right)\left(\mathbf{I} + \tfrac{\Delta t}{2}\boldsymbol{\gamma}\right)^{-1} \tag{4.35}$$

$$\mathbf{f}^t = \mathbf{M}^{-1}\mathbf{F}^t, \quad \mathbf{f}^{t+\frac{\Delta t}{2}} = \mathbf{M}^{-1}\mathbf{F}^{t+\frac{\Delta t}{2}}, \quad \mathbf{f}^{t+\Delta t} = \mathbf{M}^{-1}\mathbf{F}^{t+\Delta t} \tag{4.36}$$

$$\boldsymbol{\theta}_t^{t+\frac{\Delta t}{2}} = \mathbf{M}^{-1}\boldsymbol{\beta}_t^{t+\frac{\Delta t}{2}}, \quad \boldsymbol{\theta}_t^{t+\Delta t} = \mathbf{M}^{-1}\boldsymbol{\beta}_t^{t+\Delta t} \tag{4.37}$$

4.2. Calculation of random force

The approximation of the random force vector was obtained from the friction matrix[13,14] as

$$\boldsymbol{\beta}_t^{t+\Delta t} = \mathbf{S}\mathbf{w}^{\Delta t} \tag{4.38}$$

where $\mathbf{w}^{\Delta t}$ is a $6\mathrm{N} \times 1$ Gaussian-distributed random vector with zero mean and variance of Δt at an instant time t, and $\mathbf{S}$ is a $6\mathrm{N} \times 6\mathrm{N}$ matrix from the Cholesky decomposition of the friction matrix as

$$\mathbf{S}\mathbf{S}^{\mathrm{T}} = 2\mathrm{k_B}\mathrm{T}\mathbf{Z} \tag{4.39}$$

The positive definiteness of the mobility matrix was proved[7,15], implying the possibility of the Cholesky decomposition.

4.3. Implementation

The time integration procedure was implemented as follows.

Algorithm: Langevin dynamics simulation

**1. Initialization** $(t = 0)$	
Mass matrix	$\mathbf{M}$
Mechanical properties	$\mathbf{G}, \mathbf{R}, \mathbf{C}$
Electrostatic properties	$\mathbf{E}$
Radius of the bead	σ
Dynamic viscosity	η
Time step	Δt
Coordinate	$\mathbf{U}$
Triad	$\mathbf{T}$
Mobility matrix	$\boldsymbol{\Xi} = \boldsymbol{\Xi}(\mathbf{U}, \sigma, \eta)$
Friction matrix	$\mathbf{Z} = \boldsymbol{\Xi}^{-1}, \quad \boldsymbol{\gamma} = \mathbf{M}^{-1}\mathbf{Z}$
Cholesky decomposition	$\mathbf{S}\mathbf{S}^{\boldsymbol{T}} = 2\mathrm{k_B T}\mathbf{Z}$
Auxiliary matrices	$\mathbf{j} \leftarrow \left(\mathbf{I} + \frac{\Delta t}{4}\boldsymbol{\gamma}\right)^{-1}, \quad \mathbf{b} \leftarrow \left(\mathbf{I} + \frac{\Delta t}{2}\boldsymbol{\gamma}\right)^{-1}$
**2. Time integration** $(t \to t + \Delta t)$	
Initial configuration (t)	
Internal force	$\mathbf{f}^t \leftarrow \mathbf{f}^t(\mathbf{U}^t, \mathbf{T}^t, \mathbf{G}, \mathbf{R}, \mathbf{C}, \mathbf{E}, \mathbf{M})$
Update configuration $(t + \frac{\Delta t}{2})$	
Random force	$\boldsymbol{\theta}_t^{t+\frac{\Delta t}{2}} \leftarrow \mathbf{M}^{-1}\mathbf{S}\mathbf{w}^{\frac{\Delta t}{2}}$
Coordinate	$\Delta\mathbf{U}^{t+\frac{\Delta t}{2}} \leftarrow \Delta t\mathbf{j}\left(\frac{1}{2}\mathbf{V}^t + \frac{\Delta t}{8}\mathbf{f}^t + \frac{1}{4}\boldsymbol{\theta}_t^{t+\frac{\Delta t}{2}}\right)$
Increment	$\mathbf{U}^{t+\frac{\Delta t}{2}} \leftarrow \mathbf{U}^t + \Delta\mathbf{U}^{t+\frac{\Delta t}{2}}$
Triad	$\mathbf{T}^{t+\frac{\Delta t}{2}} \leftarrow \mathbf{T}^{t+\frac{\Delta t}{2}}\left(\mathbf{U}^{t+\frac{\Delta t}{2}}, \mathbf{T}^t\right)$
Internal force	$\mathbf{f}^{t+\frac{\Delta t}{2}} \leftarrow \mathbf{f}^{t+\frac{\Delta t}{2}}\left(\mathbf{U}^{t+\frac{\Delta t}{2}}, \mathbf{T}^{t+\frac{\Delta t}{2}}, \mathbf{G}, \mathbf{R}, \mathbf{C}, \mathbf{E}, \mathbf{M}\right)$
Update configuration $(t + \Delta t)$	
Random force	$\boldsymbol{\theta}_t^{t+\Delta t} \leftarrow \mathbf{M}^{-1}\mathbf{S}\mathbf{w}^{\Delta t}$
Increment	$\Delta\mathbf{U}^{t+\Delta t} \leftarrow \Delta t\mathbf{b}\left(\mathbf{V}^t + \frac{\Delta t}{2}\mathbf{f}^{t+\frac{\Delta t}{2}} + \frac{1}{2}\boldsymbol{\theta}_t^{t+\Delta t}\right)$
Coordinate	$\mathbf{U}^{t+\Delta t} \leftarrow \mathbf{U}^t + \Delta\mathbf{U}^{t+\Delta t}$
Triad	$\mathbf{T}^{t+\Delta t} \leftarrow \mathbf{T}^{t+\Delta t}\left(\mathbf{U}^{t+\Delta t}, \mathbf{T}^t\right)$
Internal force	$\mathbf{f}^{t+\Delta t} \leftarrow \mathbf{f}^{t+\Delta t}\left(\mathbf{U}^{t+\Delta t}, \mathbf{T}^{t+\Delta t}, \mathbf{G}, \mathbf{R}, \mathbf{C}, \mathbf{E}, \mathbf{M}\right)$
Velocity	$\mathbf{V}^{t+\Delta t} \leftarrow \mathbf{V}^{t+\Delta t}\left(\mathbf{U}^t, \mathbf{U}^{t+\Delta t}, \mathbf{V}^t, \mathbf{f}^t, \mathbf{f}^{t+\frac{\Delta t}{2}}, \mathbf{f}^{t+\Delta t}, \boldsymbol{\theta}_t^{t+\Delta t}, \boldsymbol{\gamma}, \Delta t\right)$
**3. End of simulation**	

4.4. Linear analysis

We computed essential statistical measures of the time integration scheme for representative linear cases in order to assess its general applicability. Here we employed the particle notation (U, V) for simplicity.

(Case I) Thermal diffusion in a flat potential

We do not need to consider the coordinate update for the half time step $\mathrm{U}^t \to \mathrm{U}^{t+\Delta t/2}$. By invoking no internal force ($\mathrm{f}^t = \mathrm{f}^{t+\Delta t/2} = 0$) into the equations (4.32) and (4.34), we obtain

$$\mathrm{U}^{t+\Delta t} = \mathrm{U}^t + \Delta t\mathrm{b}\left(\mathrm{V}^t + \frac{\Delta t}{2}\mathrm{f}^{t+\frac{\Delta t}{2}} + \frac{1}{2}\theta_t^{t+\Delta t}\right) = \mathrm{U}^t + \Delta t\mathrm{b}\mathrm{V}^t + \frac{1}{2}\Delta t\mathrm{b}\theta_t^{t+\Delta t} \tag{4.40}$$

$$\mathrm{V}^{t+\Delta t} = a\mathrm{V}^t + \frac{\Delta t}{6}\left(\mathrm{f}^{t+\Delta t} + 2(a+b)\mathrm{f}^{t+\frac{\Delta t}{2}} + \mathrm{f}^t\right) + \mathrm{b}\theta_t^{t+\Delta t} = a\mathrm{V}^t + \mathrm{b}\theta_t^{t+\Delta t} \tag{4.41}$$

These two updating relations are exactly the same as in the original GJF scheme[13]. Therefore, the resulting velocity distribution from the equation (4.41) reproduces a zero mean Gaussian (Maxwell-Boltzmann) distribution with the deviation as

$$\langle(\mathrm{V^t})^2\rangle = \frac{\mathrm{k_B T}}{\mathrm{m}} \tag{4.42}$$

which leads to an average kinetic energy of

$$\langle \mathrm{E_k}\rangle = \frac{1}{2}\mathrm{m}\langle(\mathrm{V^t})^2\rangle = \frac{\mathrm{k_B T}}{2} \tag{4.43}$$

The coordinate relation is also transformed into the diffusion matrix as

$$\mathrm{D} = \lim_{n\to\infty} = \frac{\langle \mathrm{U}^{t+n\Delta t} - \mathrm{U}^0\rangle^2}{2n\Delta t} = \frac{\mathrm{k_B T}}{\zeta} \tag{4.44}$$

where ζ is the friction coefficient. This gives the correct expectation for coordinate and velocity distribution for any parameter including the time step.

(Case II) Thermal harmonic oscillator

Introducing the harmonic potential ($\mathrm{f}^t = -k\mathrm{U}^t$, $\mathrm{f}^{t+\Delta t/2} = -k\mathrm{U}^{t+\Delta t/2}$, $\mathrm{f}^{t+\Delta t} = -k\mathrm{U}^{t+\Delta t}$), we obtain the coordinate and velocity relations as

$$\begin{bmatrix}\mathrm{U}^{t+\Delta t}\\ \mathrm{V}^{t+\Delta t}\end{bmatrix} = \mathbf{A}\begin{bmatrix}\mathrm{U}^t\\ \mathrm{V}^t\end{bmatrix} + \mathbf{N}\theta_t^{t+\Delta t} + \mathbf{M}\theta_t^{t+\frac{\Delta t}{2}} \tag{4.45}$$

where

$$\mathbf{A} = \begin{bmatrix}A_{11} & A_{12}\\ A_{21} & A_{22}\end{bmatrix}, \qquad \mathbf{N} = \begin{bmatrix}N_1\\ N_2\end{bmatrix}, \qquad \mathbf{M} = \begin{bmatrix}M_1\\ M_2\end{bmatrix} \tag{4.46}$$

with $\Omega = \sqrt{k/m}$ is natural frequency of the oscillator and

$$\begin{aligned} A_{11} &= 1 - \mathrm{b}\frac{\Omega^2\Delta t^2}{2}\left(1 - \mathrm{j}\frac{\Omega^2\Delta t^2}{8}\right)\\ A_{12} &= \mathrm{b}\Delta t\left(1 - \mathrm{j}\frac{\Omega^2\Delta t^2}{4}\right)\\ A_{21} &= -\mathrm{b}\Omega^2\Delta t\left(1 - \frac{\Omega^2\Delta t^2}{12}\right) + \mathrm{bj}\frac{\Omega^4\Delta t^3}{8}\left(1 - \frac{\Omega^2\Delta t^2}{12}\right) - \mathrm{j}\frac{\Omega^4\Delta t^3}{24}\\ A_{22} &= a - \mathrm{b}\frac{\Omega^2\Delta t^2}{6} - \mathrm{bj}\frac{\Omega^2\Delta t^2}{2}\left(1 - \mathrm{bj}\frac{\Omega^2\Delta t^2}{12}\right) + \mathrm{j}\frac{\Omega^2\Delta t^2}{6} \end{aligned} \tag{4.47}$$

$$N_1 = \mathrm{b}\frac{\Delta t}{2}, \qquad N_2 = \mathrm{b}\left(1 - \frac{\Omega^2 \Delta t^2}{12}\right)$$
$$M_1 = -\mathrm{bj}\frac{\Delta t^3}{8}, \qquad M_2 = -\mathrm{j}\frac{\Omega^2 \Delta t^2}{12}\left(\mathrm{a} + \mathrm{b} - \mathrm{b}\frac{\Omega^2 \Delta t^2}{4}\right)$$

To analze the stability region, we drived the eigenvalues of the matrix $\mathbf{A}$ as

$$\Lambda_\pm = \lambda_1 \pm \sqrt{\lambda_1^2 - \lambda_2} \tag{4.48}$$

from the characteristic equation

$$\Lambda^2 - (A_{11} + A_{22})\Lambda + (A_{11}A_{22} - A_{12}A_{21}) = \Lambda^2 - 2\boldsymbol{\lambda_1}\Lambda + \boldsymbol{\lambda_2} = 0 \tag{4.49}$$

with

$$\begin{aligned} \boldsymbol{\lambda_1} &= \mathrm{b}\left(1 - \frac{\Omega^2 \Delta t^2}{3}\right) - \mathrm{bj}\frac{\Omega^2 \Delta t^2}{4}\left(1 - \frac{5\Omega^2 \Delta t^2}{24}\right) + \mathrm{j}\frac{\Omega^2 \Delta t^2}{12} \\ \boldsymbol{\lambda_2} &= a + \mathrm{b}\frac{\Omega^2 \Delta t^2}{3} - \mathrm{bj}\frac{\Omega^2 \Delta t^2}{2}\left(1 - \frac{\Omega^2 \Delta t^2}{8}\right) + \mathrm{j}\frac{\Omega^2 \Delta t^2}{6} \end{aligned} \tag{4.50}$$

We employed $\Omega\Delta t/2$ and $\gamma/2\Omega$ as two characteristic parameters of a thermal harmonic oscillator. The four regions of characteristic behavior as a function of those two parameters are numerically demonstrated (A, B, C, and D in Supplementary Figure 29) as below.

(A) $\Lambda_\pm$ are complex (Underdamping)

(B) $\Lambda_\pm$ are real and $|\Lambda_\pm| < 1$ (Overdamping)

(C) $\Lambda_\pm$ are real and $\Lambda_+\Lambda_- < 1$

(D) $\Lambda_\pm$ are real and $|\Lambda_\pm| > 1$ (Numerically unstable)

Also, to investigate the correct distribution of configuration and velocity, we tested the two algorithms using four different example conditions (i, ii, iii, and iv conditions for the GJF and present algorithm in Supplementary Figure 29) as below.

(i) $[\mathrm{m},\ \mathrm{k},\ \gamma,\ \Delta t,\ \Omega\Delta t/2,\ \gamma/2\Omega] = [1.0, 1.0, 1.0, 1.0, 0.50, 0.50]$

(ii) $[\mathrm{m},\ \mathrm{k},\ \gamma,\ \Delta t,\ \Omega\Delta t/2,\ \gamma/2\Omega] = [1.0, 1.0, 3.0, 0.5, 0.25, 1.50]$

(iii) $[\mathrm{m},\ \mathrm{k},\ \gamma,\ \Delta t,\ \Omega\Delta t/2,\ \gamma/2\Omega] = [1.0, 1.0, 3.0, 1.5, 0.75, 1.50]$

(iv) $[\mathrm{m},\ \mathrm{k},\ \gamma,\ \Delta t,\ \Omega\Delta t/2,\ \gamma/2\Omega] = [1.0, 1.0, 3.0, 3.0, 1.50, 1.50]$

Numerical results were demonstrated in Supplementary Figure 30.

The numerical tests showed the performance of the proposed algorithm. First, the (exact) Gaussian distributions for coordinate and velocity were calculated (black lines in Supplementary Figure 30), given by

$$\mathrm{N}(\mathrm{U}) = \frac{1}{\sigma_\mathrm{U}\sqrt{2\pi}}\exp\left(-\mathrm{U}^2/2\sigma_\mathrm{U}^2\right), \qquad \sigma_\mathrm{U} = \sqrt{\mathrm{k_B T/k}} \tag{4.51}$$

$$\mathrm{N}(\mathrm{V}) = \frac{1}{\sigma_\mathrm{V}\sqrt{2\pi}}\exp\left(-\mathrm{U}^2/2\sigma_\mathrm{V}^2\right), \qquad \sigma_\mathrm{V} = \sqrt{\mathrm{k_B T/m}} \tag{4.52}$$

It was reported that the on-site velocity (V^t) of the GJF algorithm showed accurate configurational but inaccurate kinetic statistics[13] as

$$\begin{aligned} \langle \mathrm{E_p} \rangle &= \frac{1}{2}\mathrm{k}\langle (\mathrm{U^t})^2 \rangle = \frac{\mathrm{k_B T}}{2} \\ \langle \mathrm{E_k} \rangle &= \frac{1}{2}\mathrm{m}\langle (\mathrm{V^t})^2 \rangle = \frac{\mathrm{k_B T}}{2}\left[1 - \frac{\Omega^2 \Delta \mathrm{t}^2}{4}\right] \end{aligned} \tag{4.53}$$

but, we also confirmed the correct kinetic distribution, which was obtained using the half-step velocity (W^t) following the previous study[16] (i, ii, and iv conditions in Supplementary Figure 30) given by

$$W^t = V^t + \frac{1}{2}\Delta t f^t \tag{4.54}$$

On the other hand, the present scheme showed the accurate kinetic distribution using the on-site velocity (i, ii, iii, and iv conditions in Supplementary Figure 30).

In the aspect of stability, the GJF scheme is numerically unstable in the region of $\Omega\Delta t/2 > 1$, so there is a hard limit of a large time step (iv condition in Supplementary Figure 30). However, in the present algorithm, we obtained a wide stable region by employing Simpson's rule in the calculation of internal force (iv condition in Supplementary Figure 30). Although the analytic derivation of the configuration and velocity distributions remains for further research, we could numerically confirm its advantages.

Note that the sampling of the configurational space is the main objective of the simulations of DNA systems, which is an overdamped case ($\gamma/2\Omega > 30$, C region in Supplementary Figure 29). Therefore, considering the stability region, numerical tests, and robust configurational sampling properties, we could choose a large time step (Δt) using the developed algorithm for a DNA system. In future studies, the proposed algorithm could be improved more efficiently by employing constrained Langevin dynamics[17].

Supplementary Note 5. Mode analysis

To explore the deformable modes of the system, we performed both principal component analysis and normal mode analysis. The former uses dynamic trajectories in a damping solvent, while the latter considers the stiffness and mass of the system in a vacuum.

5.1. Principal component analysis

Principal component analysis was performed to extract effective vibrational modes from molecular fluctuations in the dynamic simulations. We collected 500-ns-long trajectories and saved snapshots every 250 ps, resulting in a total of 2000 frames for the pointer (v2) structure[18]. We represented the node information in a $6\mathrm{N} \times \mathrm{M}$ matrix denoted by $\mathbf{x}$, where N is the node number and M is the number of frames.

Assuming the quasi-harmonic energy[19], where each mode is populated with the energy of $\mathrm{k_B T}$, the energy function is expressed as $\Pi = \mathbf{x}^{\mathrm{T}}\mathbf{Kx/2}$. Here, the effective stiffness matrix ($\mathbf{K}$) is driven by $\mathbf{K} = \mathrm{k_B T}\boldsymbol{\sigma}^{-1}$, where $\mathrm{k_B}$ is the Boltzmann constant, T is the absolute temperature, and $\boldsymbol{\sigma}$ indicates the fluctuation matrix. The principal modes are derived using the $6\mathrm{N} \times 6\mathrm{N}$ fluctuation matrix, given by

$$\boldsymbol{\sigma} = \langle(\mathbf{x} - \langle\mathbf{x}\rangle)(\mathbf{x} - \langle\mathbf{x}\rangle)^{\mathrm{T}}\rangle \qquad (5.1)$$

where the angle bracket represents the time average. The mass-weighted fluctuation matrix ($\boldsymbol{\Sigma}$) is then calculated using the global mass matrix ($\mathbf{M}$) as

$$\boldsymbol{\Sigma} = \mathbf{M}^{1/2}\boldsymbol{\sigma}\mathbf{M}^{1/2} \qquad (5.2)$$

Finally, we obtained the eigenmodes of the structure by performing normal mode analysis as

$$\boldsymbol{\Sigma\Phi} = \boldsymbol{\Phi\Lambda} \qquad (5.3)$$

thereby providing the set of eigenvectors ($\boldsymbol{\Phi} = [\boldsymbol{\phi}_1 \quad \boldsymbol{\phi}_2 \quad \cdots]$) and eigenvalues ($\boldsymbol{\Lambda} = [\lambda_1 \quad \lambda_2 \quad \cdots]$)). To investigate the lowest modes, we computed the ten largest eigenvalues and corresponding eigenvectors. The natural frequencies (ω_i) and mode shapes ($\Delta\mathbf{x}_{\mathrm{i}}$) are given by

$$\omega_i = (\mathrm{k_B T}/\Lambda_i)^{1/2} \qquad (5.4)$$

$$\Delta\mathbf{x}_{\mathrm{i}} = \left(\mathbf{M}^{1/2}\right)^{-1}\boldsymbol{\phi}_i \qquad (5.5)$$

5.2. Normal mode analysis

Normal mode analysis was performed for the final configuration of dynamic simulations. We considered the eigenvalue equation given by

$$\mathbf{K\Phi} = \mathbf{M\Phi\Lambda} \qquad (5.6)$$

where $\mathbf{K}$ and $\mathbf{M}$ represent the global stiffness and mass matrices, $\boldsymbol{\Phi}$ and $\boldsymbol{\Lambda}$ are the eigenvectors ($\boldsymbol{\Phi} = [\boldsymbol{\phi}_1 \quad \boldsymbol{\phi}_2 \quad \cdots]$) and corresponding eigenvalues ($\boldsymbol{\Lambda} = [\lambda_1 \quad \lambda_2 \quad \cdots]$). We calculated the smallest ten eigenmodes to observe the lowest modes. The natural frequencies and mode shapes are given as

$$\omega_i = (\Lambda_i)^{1/2} \qquad (5.7)$$

$$\Delta \mathbf{x}_{\mathrm{i}} = \left(\mathbf{M}^{1/2}\right)\boldsymbol{\phi}_i \tag{5.8}$$

Supplementary Note 6. Stacking model

We considered the stacking interaction between distant base-pairs. The Morse potential energy function was employed to model the stacked and unstacked base-pairs[20,21]. The potential depends only on the distance, so the spring finite element model was implemented like the electrostatic interaction[2].

6.1. Stacking energy

The stacking energy (Π_{SK}) with a distance (r) was modeled using the Morse potential by

$$\Pi_{\mathrm{SK}}(\mathrm{r}) = \varepsilon\left[1 - \exp\left(-\mathrm{a}(\mathrm{r} - \mathrm{r}_0)\right)\right]^2 - \varepsilon \tag{6.1}$$

where ε is the energy parameter for dissociation of stacking, a is the shape parameter, and r_0 is the equilibrium distance, respectively. The energy value at an infinite distance goes to zero.

The model parameters (ε, a, and r_0) were fitted to the potential of mean force (PMF) derived from the trajectories of the stacking distances in MD simulations as

$$\Pi_{\mathrm{PMF}}(\mathrm{r}) = -\mathrm{k_B T} \log[\mathrm{g}(\mathrm{r})] \tag{6.2}$$

where $\mathrm{g}(\mathrm{r})$ represents the radial distribution function, computed by the normalized histogram of the stacking distances divided by $4\pi\mathrm{r}^2$.

6.2. Finite element model

The stacking energy is a function of the distance between two nodes as $\Pi_{\mathrm{SK}} = \Pi_{\mathrm{SK}}(\mathrm{r})$, without considering the rotational degrees of freedom. This indicates that the stacking interaction can be modeled as an equivalent spring element whose length is the distance between two nodes.

We derived the stiffness matrix and the internal force vector of the spring element by considering the axial deformation only. Note that only the internal force vector is used in the dynamic simulations, but we derived a stiffness matrix for complete modeling. The axial extension of the spring was considered for the prismatic and homogeneous spring with the initial length ($\mathrm{r_i}$) and the stretched length ($\mathrm{r_f}$) at the incremented step as follows.

$$\mathrm{r_i} = \left|{}^{\mathrm{i}}\vec{\mathrm{u}}_2 - {}^{\mathrm{i}}\vec{\mathrm{u}}_1\right| \quad \mathrm{r_f} = \left|{}^{\mathrm{f}}\vec{\mathrm{u}}_2 - {}^{\mathrm{f}}\vec{\mathrm{u}}_1\right| \tag{6.3}$$

where ${}^{i}\vec{\mathrm{u}}_n$ and ${}^{f}\vec{\mathrm{u}}_n$ represent the 3×1 coordinate vectors with only translation terms ($\mathrm{n} = 1,2$), respectively as

$${}^{\mathrm{i}}\vec{\mathrm{u}}_{\mathrm{n}} = [{}^{\mathrm{i}}\mathrm{x_n} \quad {}^{\mathrm{i}}\mathrm{y_n} \quad {}^{\mathrm{i}}\mathrm{z_n}]^{\mathrm{T}}, \quad {}^{\mathrm{f}}\vec{\mathrm{u}}_{\mathrm{n}} = [{}^{\mathrm{f}}\mathrm{x_n} \quad {}^{\mathrm{f}}\mathrm{y_n} \quad {}^{\mathrm{f}}\mathrm{z_n}]^{\mathrm{T}} \tag{6.4}$$

First, the Green-Lagrange strain of the spring element (e) was given as

$$\mathrm{e} = \frac{\mathrm{r_f^2} - \mathrm{r_i^2}}{2\mathrm{r_i^2}} \tag{6.5}$$

Next, the second Piola-Kirchhoff stress (S) of the spring element was derived as the standard stress measure of the Green-Lagrange strain as

$$\mathrm{S} = \frac{\mathrm{r_i}}{\mathrm{r_f}}\frac{1}{\mathrm{A_{ST}}}\frac{\partial \Pi_{\mathrm{ST}}(\mathrm{r_f})}{\partial \mathrm{r_f}} \tag{6.6}$$

where A_{ST} represents a virtual constant area and

$$\frac{\partial \Pi_{SK}(r_f)}{\partial r_f} = 2a\varepsilon \exp\left(-a(r_f - r_0)\right)\left[1 - \exp\left(-a(r_f - r_0)\right)\right] \tag{6.7}$$

The tangent elastic modulus (E), defined as the gradient of the second Piola-Kirchhoff stress for the Green-Lagrange strain, is given by

$$E = \frac{\partial S}{\partial e} = \frac{2a\varepsilon r_i^3}{A_{ST} r_f^3} \exp\left(-a(r_f - r_0)\right)\left[(1 + 2ar_f)\exp\left(-a(r_f - r_0)\right) - ar_f - 1\right] \tag{6.8}$$

Accordingly, the 12-by-12 global stiffness matrix ($\mathbf{K}_G^{SK}$) is computed by the sum of material and geometric terms. Noting that the spring element does not contain rotational degrees of freedom when assembling the global stiffness matrix, rotational components are zero values.

$$\mathbf{K}_G^{SK} = K\mathbf{P}_M + \frac{F}{r_f}\mathbf{P}_G \tag{6.9}$$

where the stiffness (K) and the force (F) terms are calculated as

$$K(r_f) = \frac{r_f^3}{r_i^3} EA_{SK} = 2a\varepsilon \exp\left(-a(r_f - r_0)\right)\left[(1 + 2ar_f)\exp\left(-a(r_f - r_0)\right) - ar_f - 1\right] \tag{6.10}$$

$$F(r_f) = \frac{r_f}{r_i} SA_{SK} = 2a\varepsilon \exp\left(-a(r_f - r_0)\right)\left[1 - \exp\left(-a(r_f - r_0)\right)\right] \tag{6.11}$$

and the matrices are given by

$$\mathbf{P}_M = \begin{bmatrix} \mathbf{p} & \mathbf{O} & -\mathbf{p} & \mathbf{O} \\ \mathbf{O} & \mathbf{O} & \mathbf{O} & \mathbf{O} \\ -\mathbf{p} & \mathbf{O} & \mathbf{p} & \mathbf{O} \\ \mathbf{O} & \mathbf{O} & \mathbf{O} & \mathbf{O} \end{bmatrix}, \mathbf{P}_G = \begin{bmatrix} \mathbf{I} & \mathbf{O} & -\mathbf{I} & \mathbf{O} \\ \mathbf{O} & \mathbf{O} & \mathbf{O} & \mathbf{O} \\ -\mathbf{I} & \mathbf{O} & \mathbf{I} & \mathbf{O} \\ \mathbf{O} & \mathbf{O} & \mathbf{O} & \mathbf{O} \end{bmatrix} \tag{6.12}$$

with

$$\mathbf{p} = \begin{bmatrix} c_x^2 & c_x c_y & c_x c_z \\ c_x c_y & c_y^2 & c_y c_z \\ c_x c_z & c_y c_z & c_z^2 \end{bmatrix}, \quad \begin{bmatrix} c_x \\ c_y \\ c_z \end{bmatrix} = \frac{1}{r_f}\begin{bmatrix} {}^f x_2 - {}^f x_1 \\ {}^f y_2 - {}^f y_1 \\ {}^f z_2 - {}^f z_1 \end{bmatrix}, \quad \mathbf{O} = \begin{bmatrix} 0 & 0 & 0 \\ 0 & 0 & 0 \\ 0 & 0 & 0 \end{bmatrix}, \quad \mathbf{I} = \begin{bmatrix} 1 & 0 & 0 \\ 0 & 1 & 0 \\ 0 & 0 & 1 \end{bmatrix} \tag{6.13}$$

The 12-by-1 internal force vector ($\vec{F}_G^{SK}$) in global coordinate is also obtained as

$$\vec{F}_G^{SK} = F\begin{bmatrix} -c_x & -c_y & -c_z & c_x & c_y & c_z \end{bmatrix}^T \tag{6.14}$$

Supplementary Figures

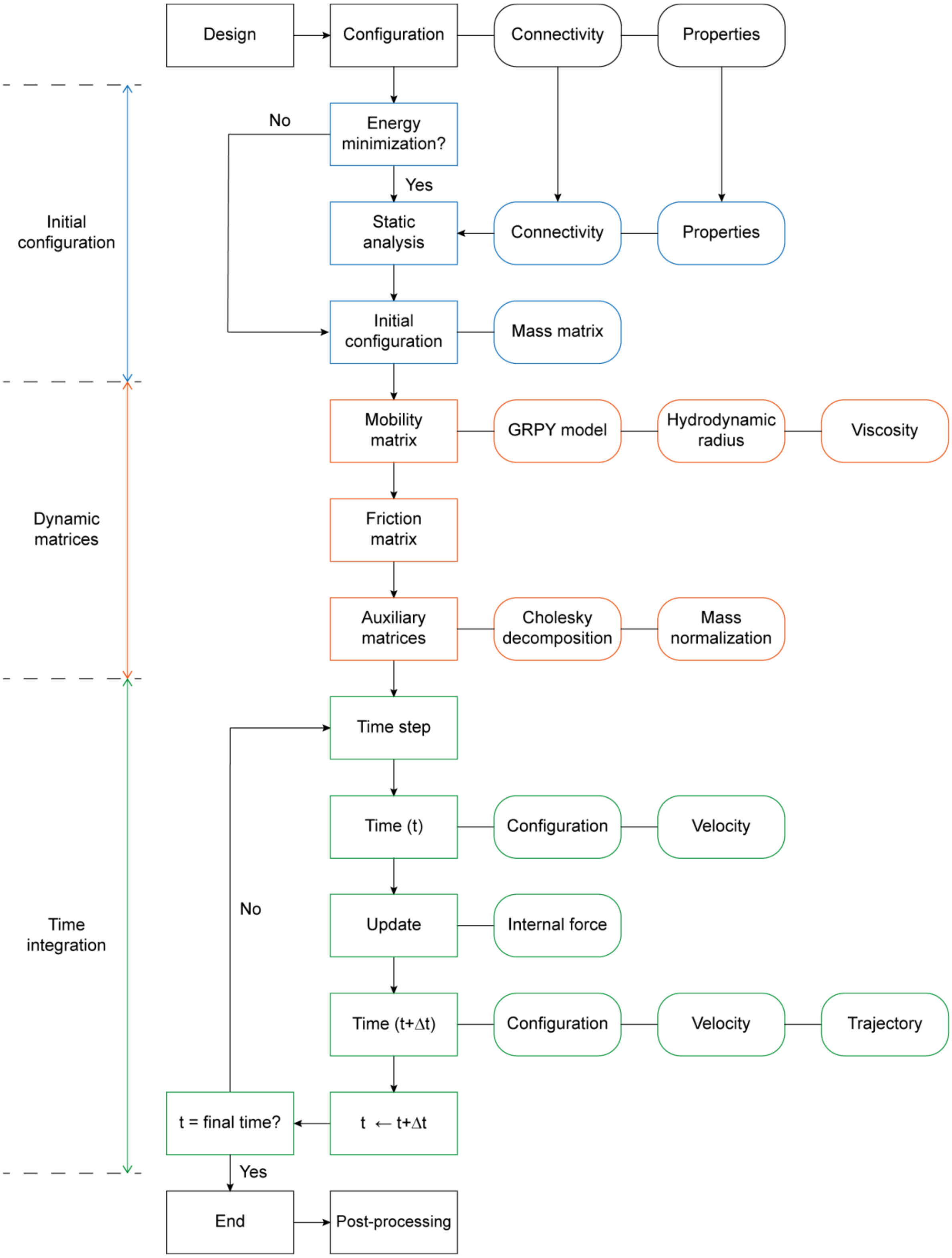

Supplementary Figure 1. Flow of the proposed dynamic framework

The framework consists of the procedure of the generation of the initial structural configuration, the generation of the hydrodynamic model, and the time integration of the Langevin equation.

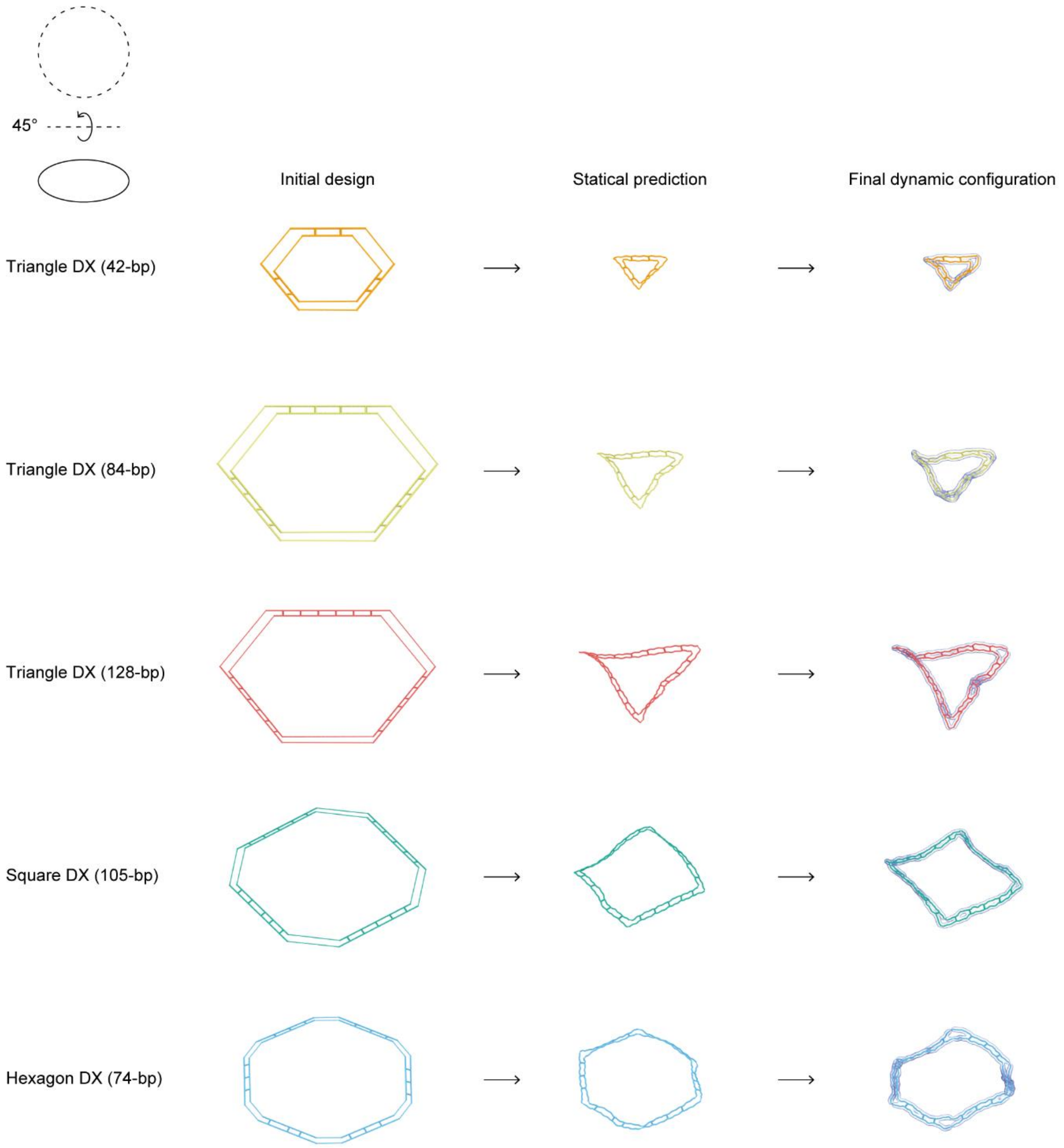

Supplementary Figure 2. Configurations of DNA wireframe structures with DX edges

For five wireframe structures with DX edges[22], the initial, statically predicted[23], and final configurations are presented.

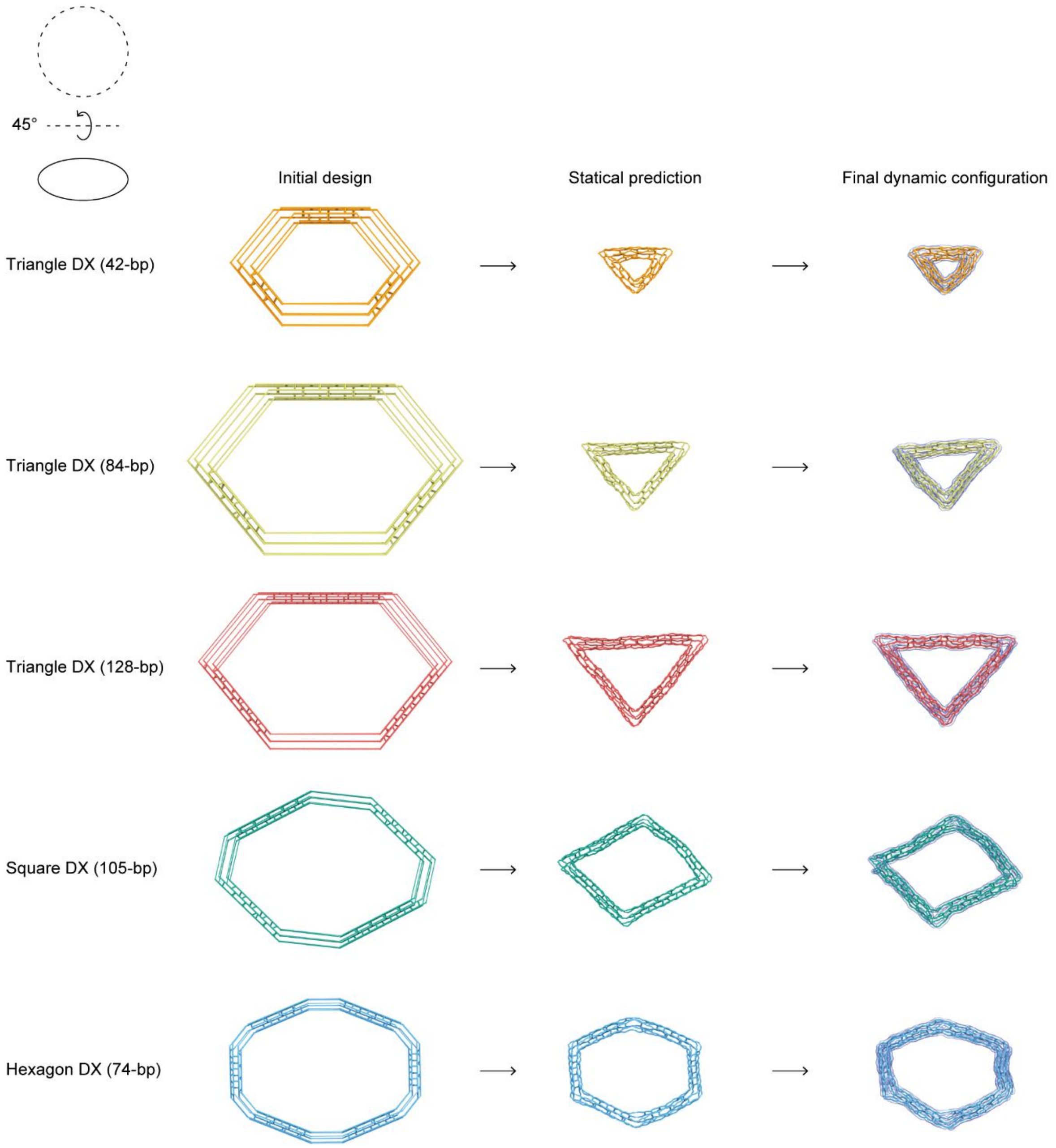

Supplementary Figure 3. Configurations of DNA wireframe structures with 6HB edges

For five wireframe structures with 6HB edges[24], the initial, statically predicted[23], and final configurations are presented.

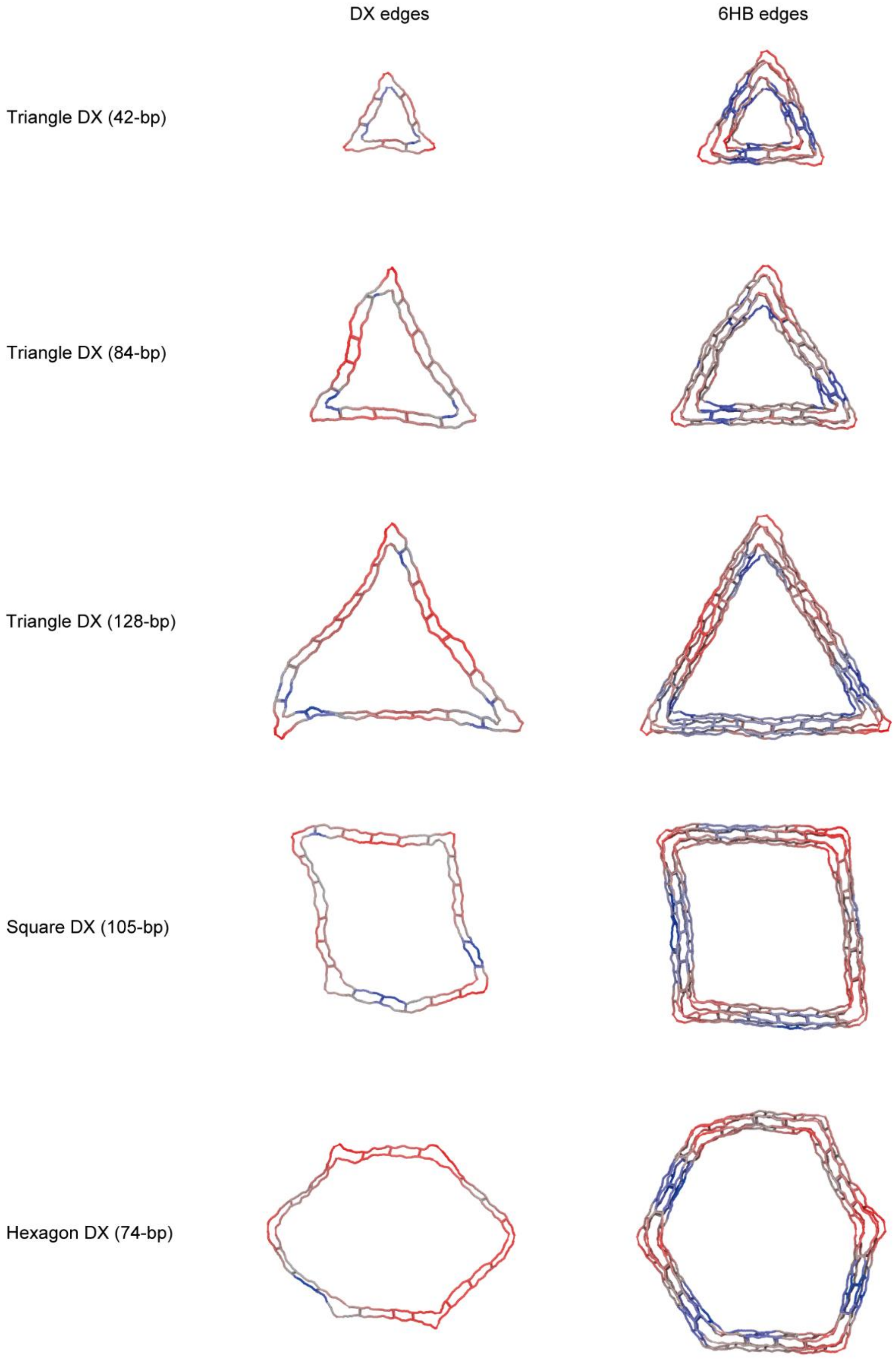

Supplementary Figure 4. RMSF distribution of DNA wireframe structures

For ten DNA wireframe structures, the RMSF distributions were computed through dynamic simulations (red: high and blue: low). The high fluctuation was observed at the vertices of the structures.

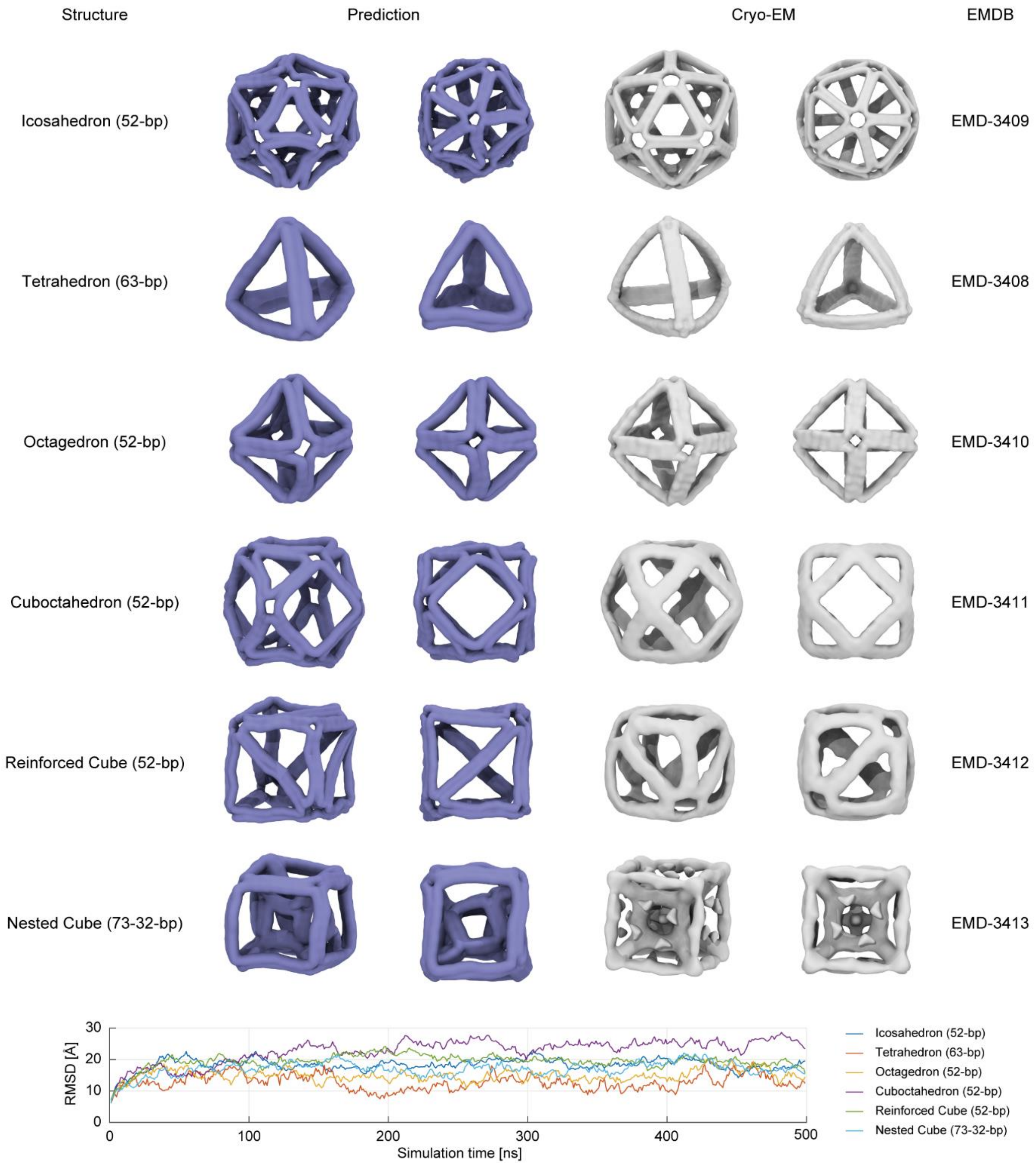

Supplementary Figure 5. Comparison of DNA wireframe structures (3D) with cryo-EM data

The six three-dimensional DNA wireframe structures (DX) were compared with cryo-EM maps[25]. For each structure, the 500-ns-long dynamic trajectory was converted into density map (violet) using VMD[26] and illustrated along with the corresponding experimental maps (white).

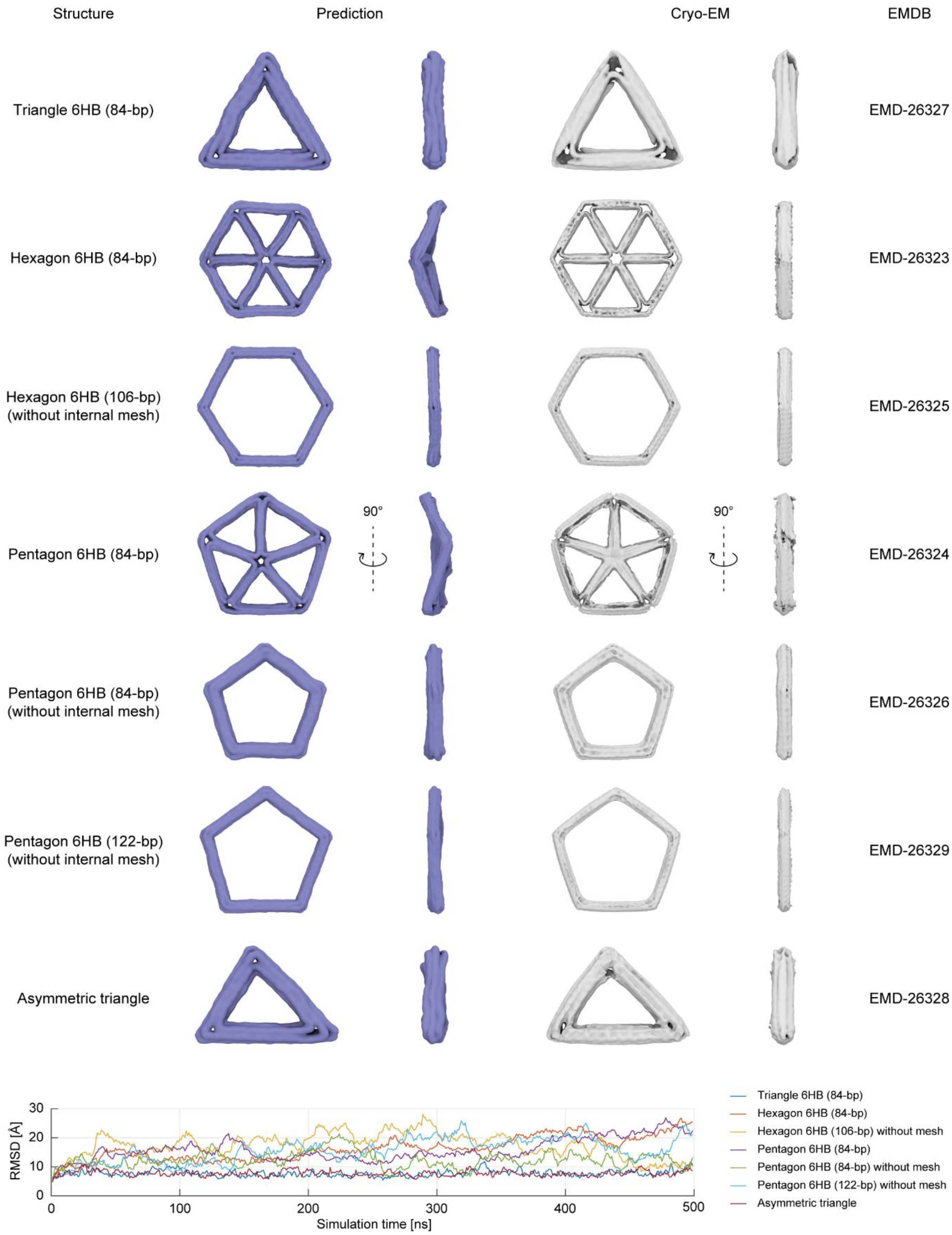

Supplementary Figure 6. Comparison of DNA wireframe structures (2D) with cryo-EM data

The seven planar DNA wireframe structures (6HB) were compared with cryo-EM maps[27]. For each structure, the 500-ns-long dynamic trajectory was converted into density map (violet) using VMD[26] and illustrated along with the corresponding experimental maps (white).

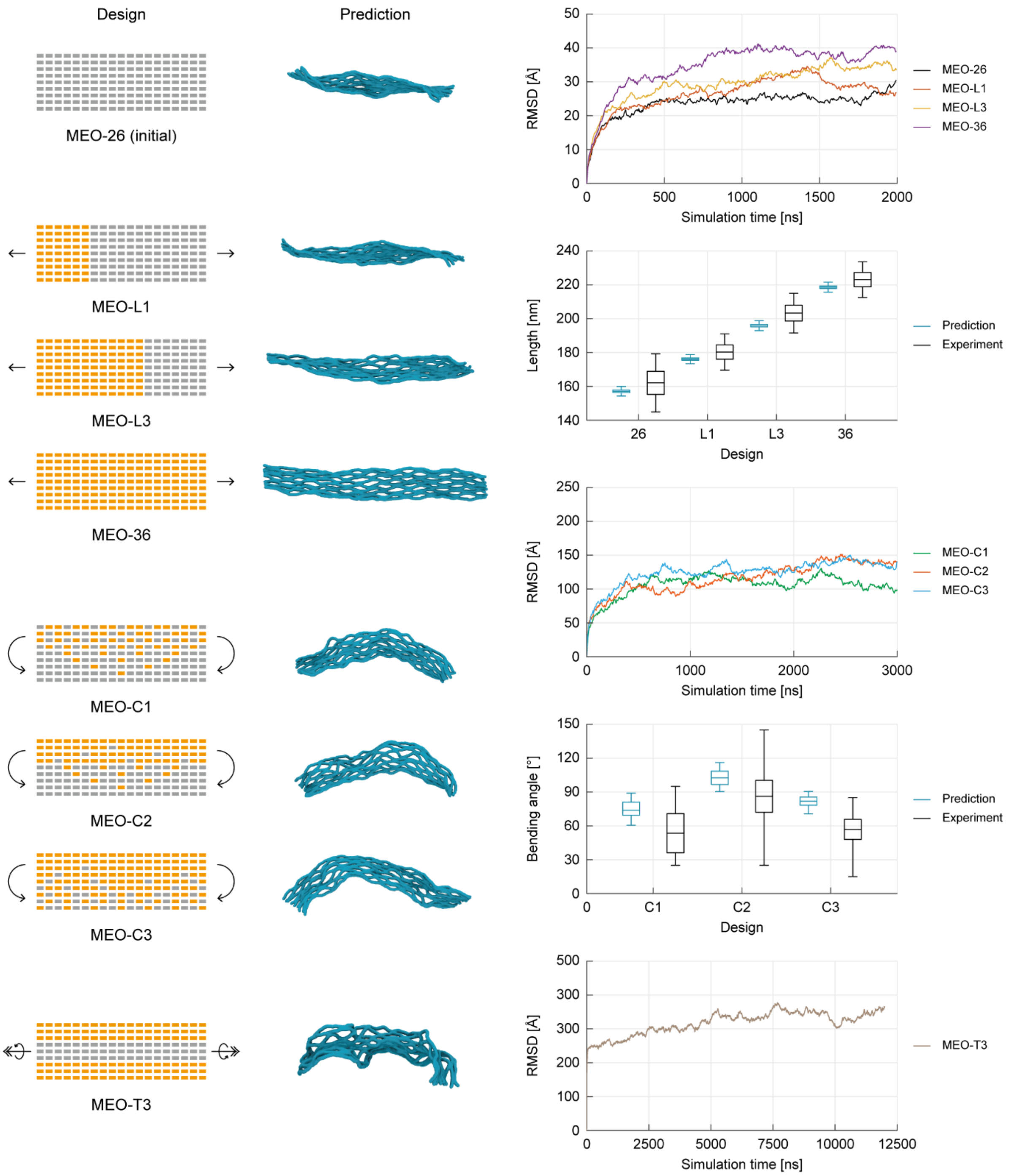

Supplementary Figure 7. Dynamic simulations of DNA structures with modular dynamic units

The DNA origami structures with modular dynamic units (modular expandable origami, MEO) [28] were predicted and compared with experiments. There were two 26-bp-long (gray) and 36-bp-long (orange) dynamic units, and different shapes of structures were programmed by controlling their combination. Since the structures are highly deformable, the static analysis was not converged. Instead, we performed dynamic simulations that began from the same initial structure (MEO-26). The length of MEO-26, MEO-L2, MEO-L3, and MEO-36 and the bending angle of MEO-C1, MEO-C2, and MEO-C2 were compared with experimental distribution (sample size: 500).

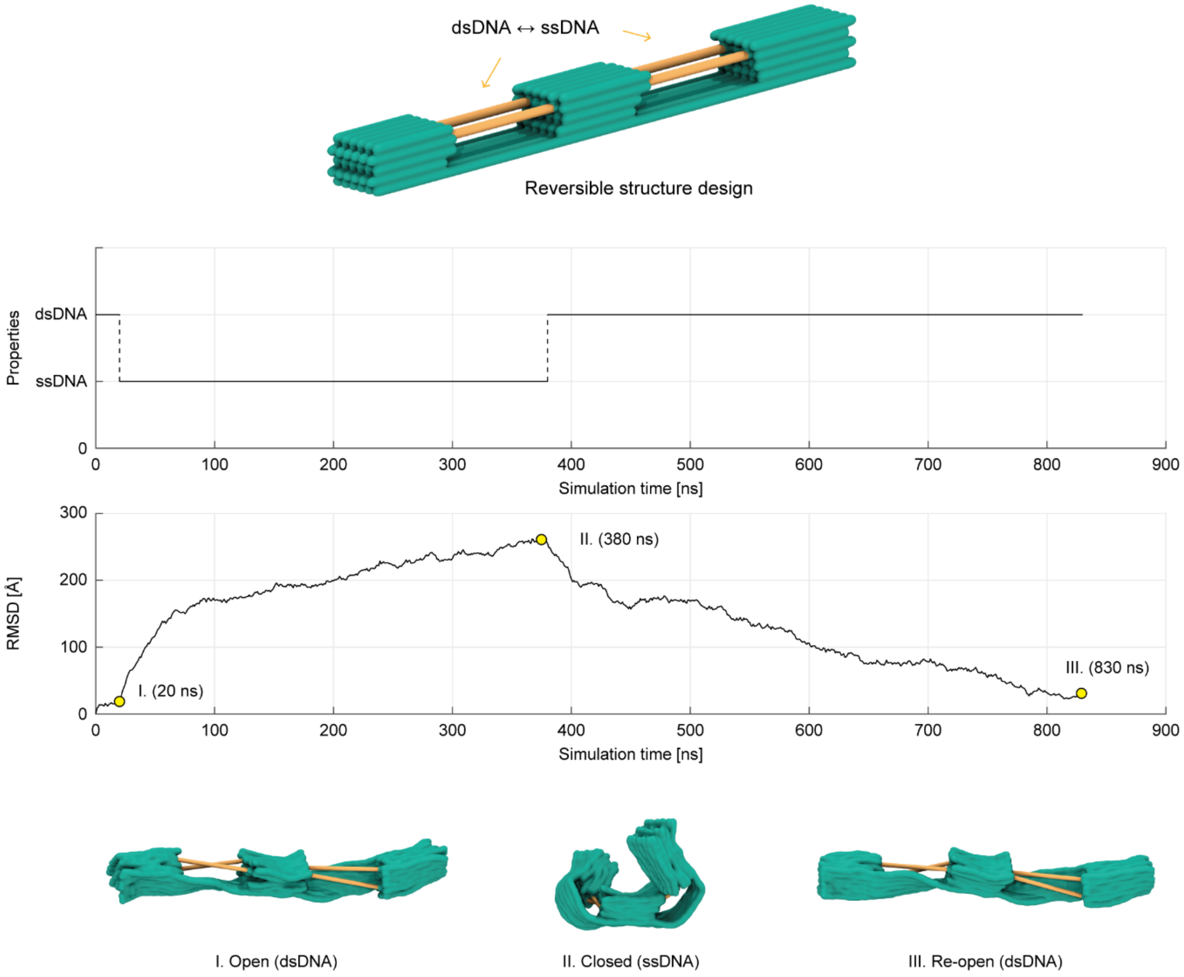

Supplementary Figure 8. Dynamic simulations of reversible DNA structures

Structural reconfiguration of the reversible DNA origami structures[29] was simulated using transitions between single-stranded DNA (ssDNA) and double-stranded DNA (dsDNA). The mechanical properties of the finite elements in the transition region (orange) were changed as dsDNA-ssDNA-dsDNA to simulate the open-closed-open reconfiguration. Initially, the geometric and mechanical properties of dsDNA were assigned to ssDNA in the transition region and the structure was maintained in an open configuration. After the RMSD curve of the open structure converged, the structure was gradually transformed into a closed configuration by assigning ssDNA properties to the transition region. Finally, the structure was reopened by reassigning dsDNA properties to the transition region.

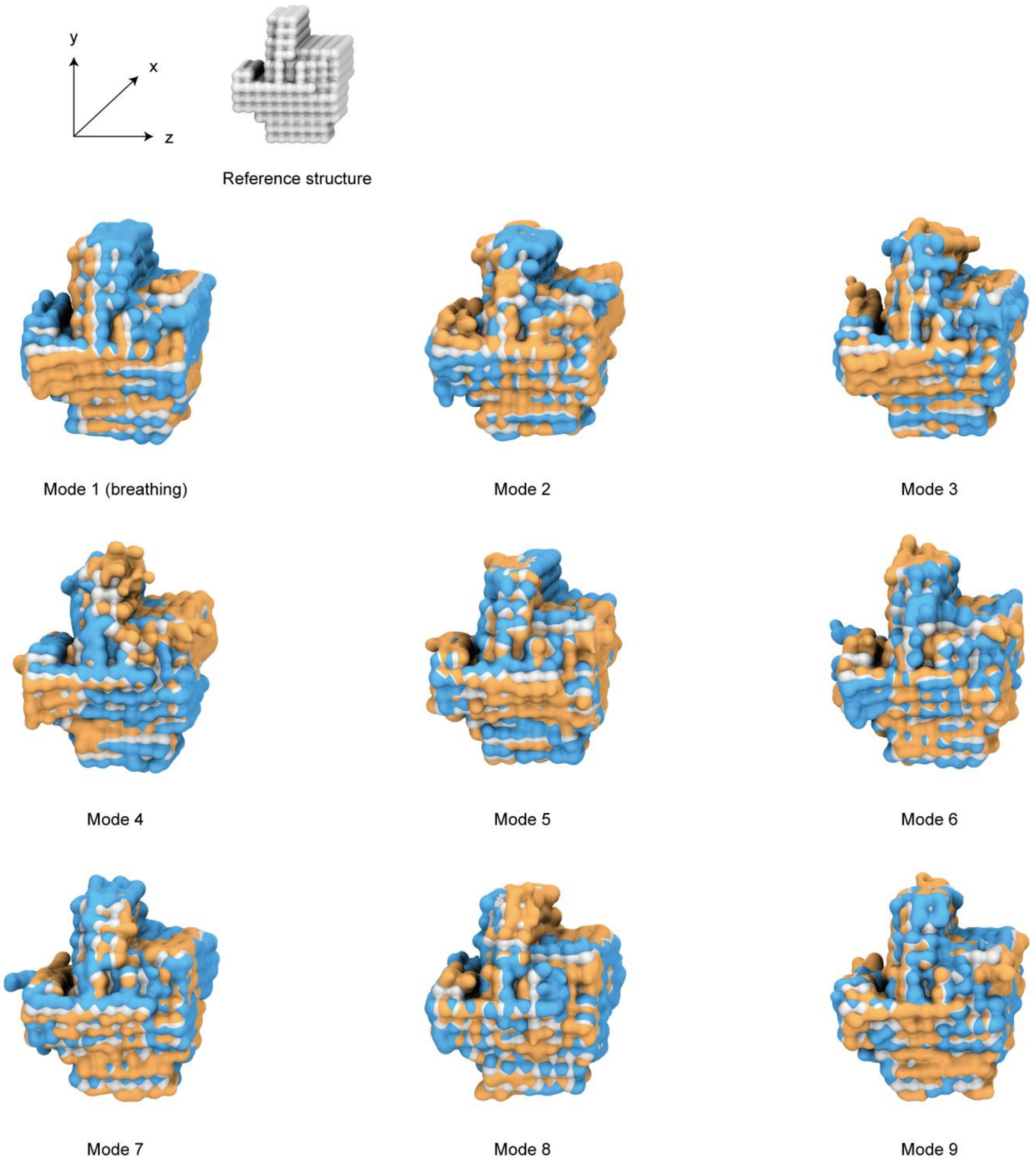

Supplementary Figure 9. Mode shapes of pointer structure using principal component analysis

Using the dynamic trajectory, the mode shapes were obtained through principal component analysis (PCA). For the reference structure (white), two mode shapes in opposite direction were illustrated (blue and orange). The rigid-body motions were removed. The breathing motion (mode 1) occurred in the direction orthogonal to the axis of constituent helices, coupled with structural rotation in a helical direction.

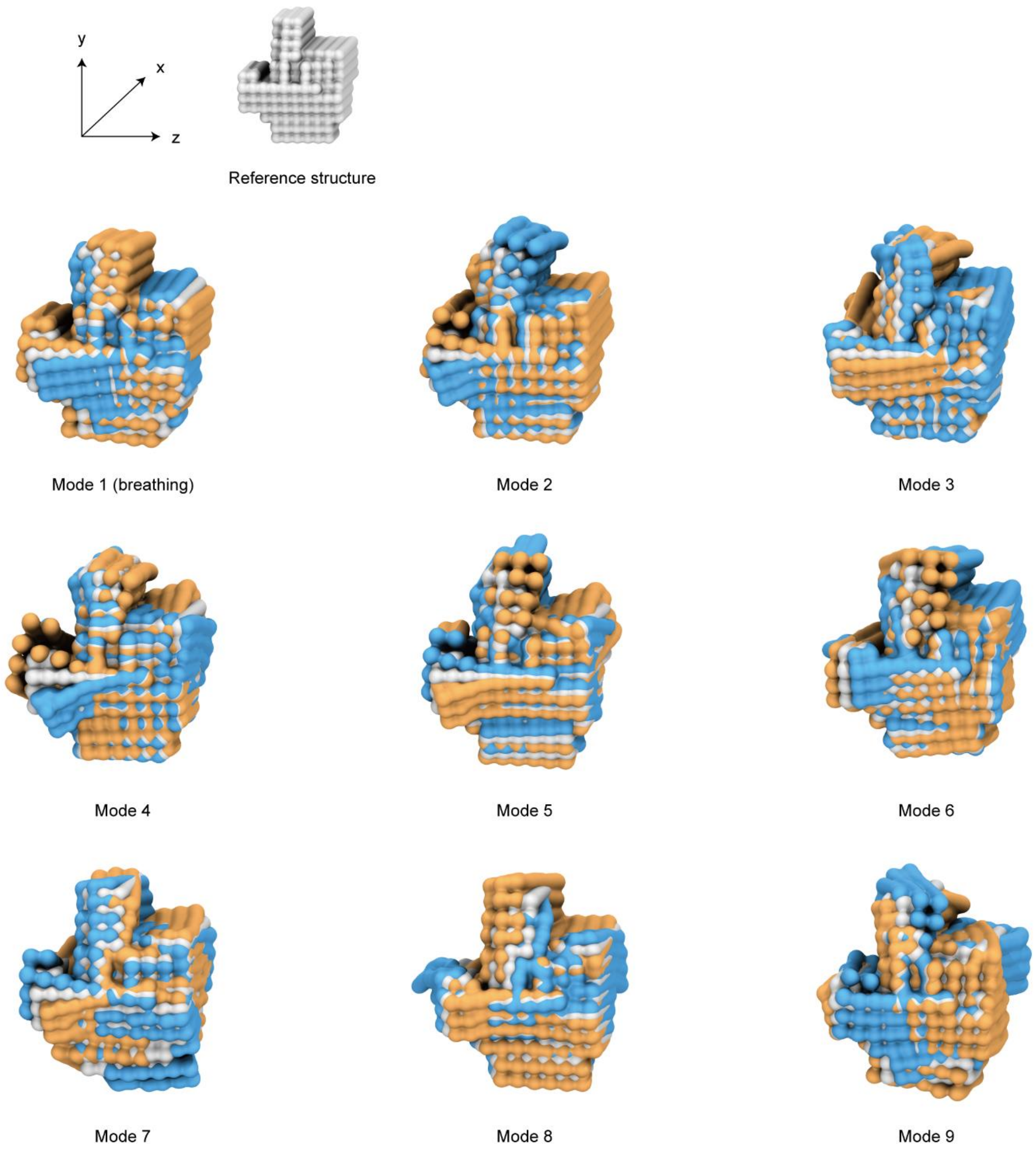

Supplementary Figure 10. Mode shapes of pointer structure using normal mode analysis

The mode shapes were obtained through normal mode analysis (NMA) in a vacuum. For the reference structure (white), two mode shapes in opposite direction were illustrated (blue and orange). The rigid-body motions were removed. The breathing motion was observed (mode 1) and coupled with structural rotation in a helical direction.

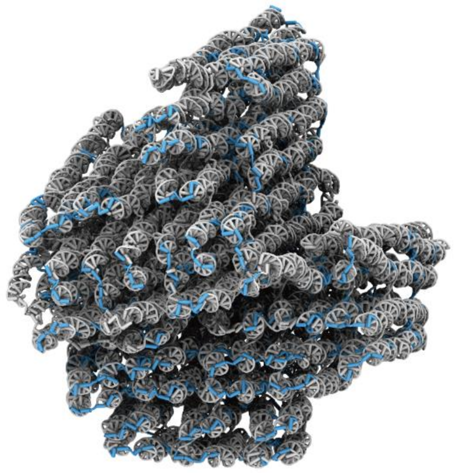

Supplementary Figure 11. The ratio of broken base-pairs in the pointer structure

The ratio of broken base-pairs for all base-pairs in the pointer structure[30] was estimated to be 5% but 18% for base-pairs at crossover sites. The pairing was considered broken when the distance of the hydrogen bond between the N1 atom of the purine base and the N3 atom of the pyrimidine base exceeded 4 Å.

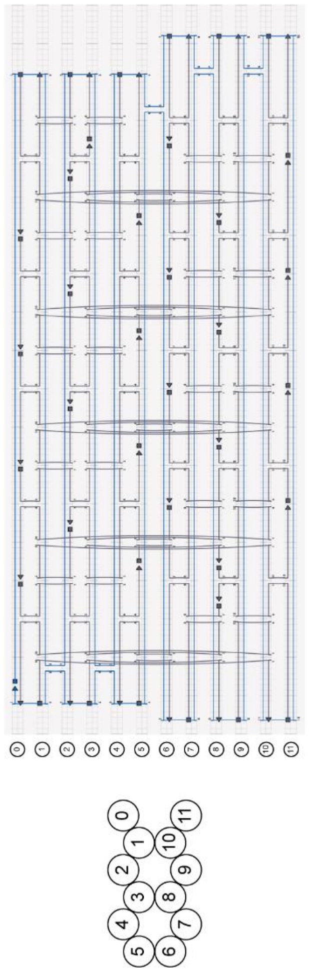

Supplementary Figure 12. Design of the 12HB structure

The fragment of the DNA origami 12-helix-bundle structure[31] was used to compare the dynamic simulations. The fragment of the DNA origami structure was used to compare the dynamic simulations. Each simulation started from an ideal (lattice) configuration from the design.

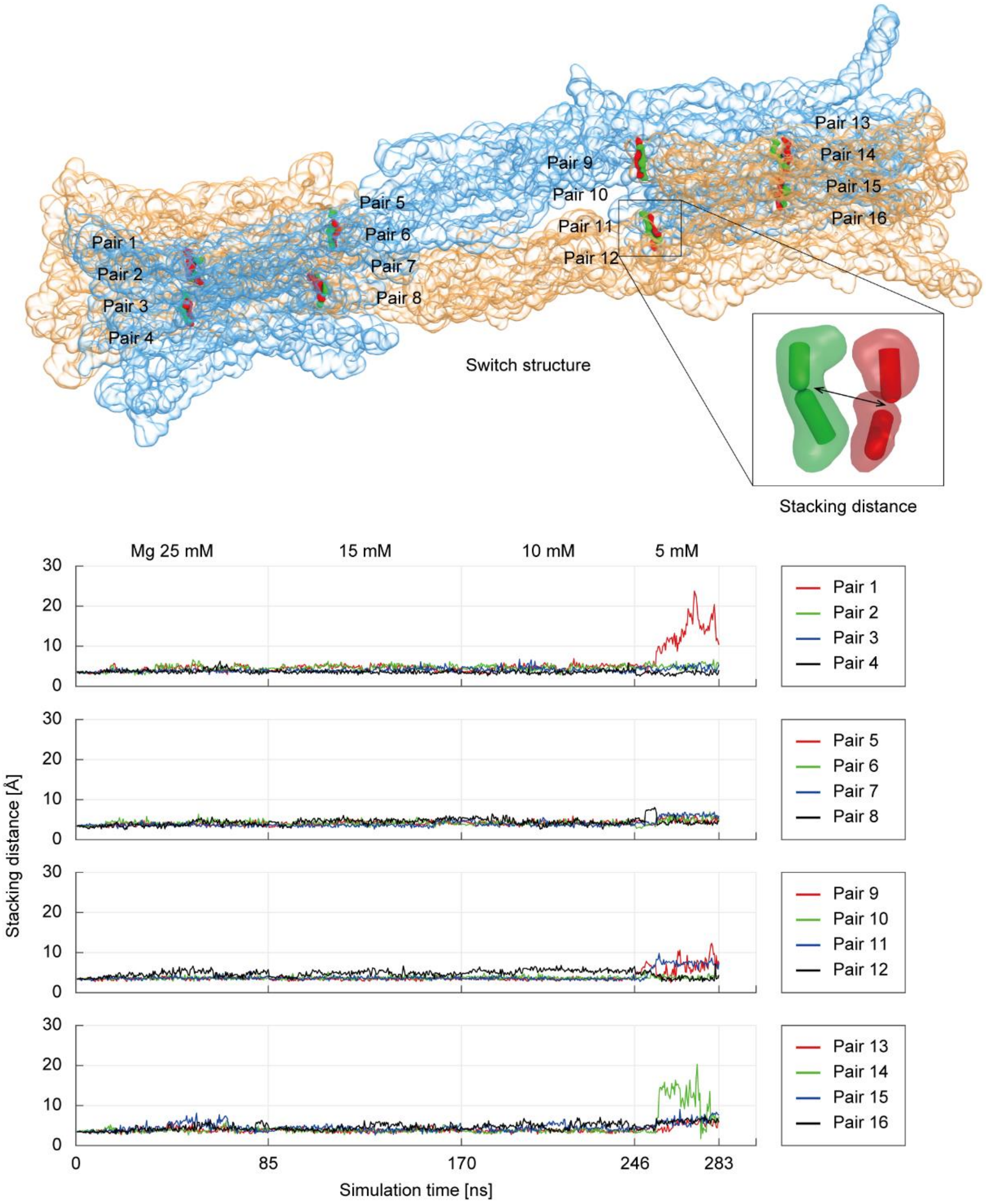

Supplementary Figure 13. Stacking distance trajectories in the switch structure

For sixteen stacking sites, the trajectories of stacking distance between two base-pairs were collected to model the stacking potential.

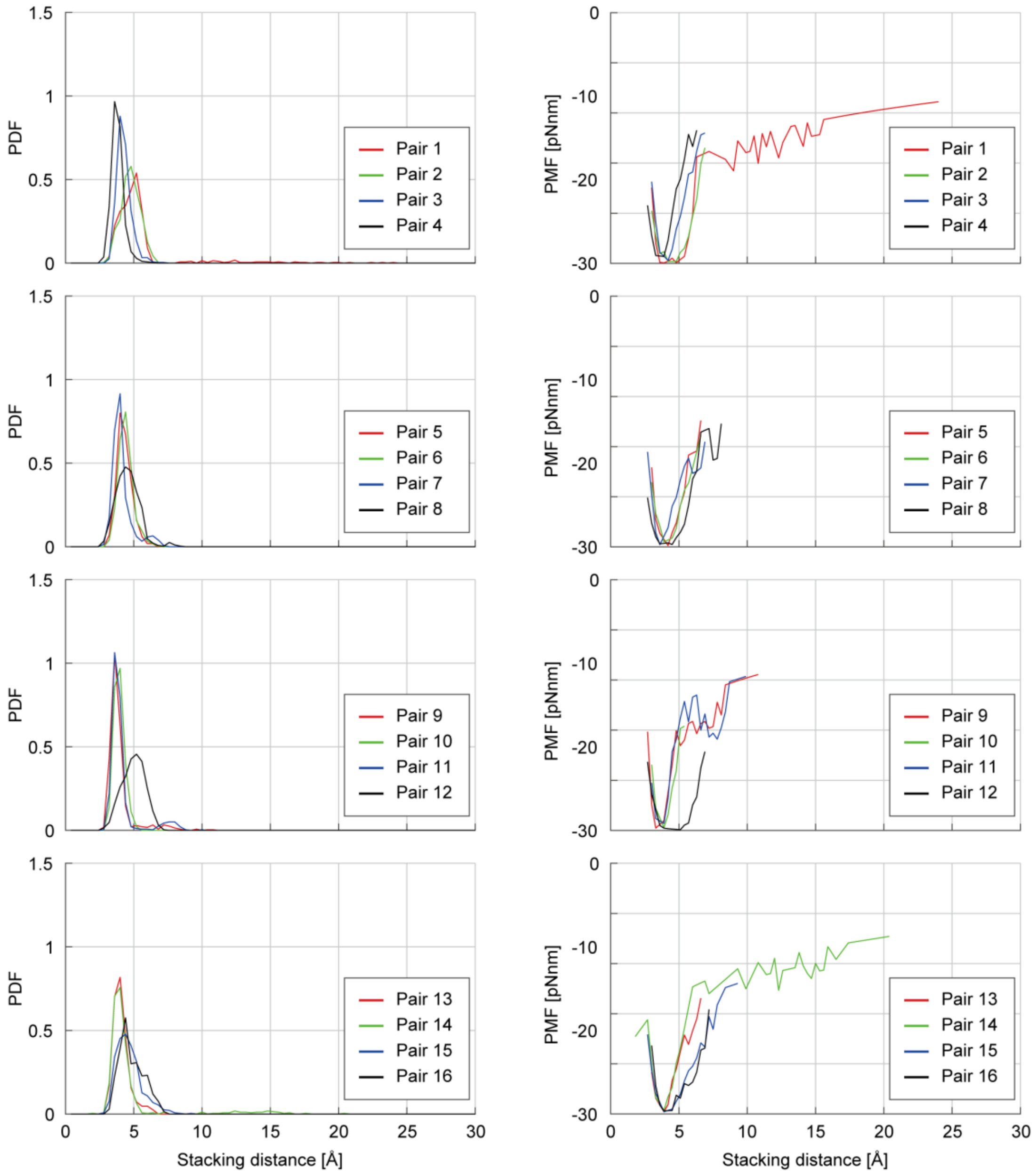

Supplementary Figure 14. Potential of mean force for stacking in the switch structure

For each stacking site, the potential of mean force (PMF) was computed to fit the model parameters of Morse potential.

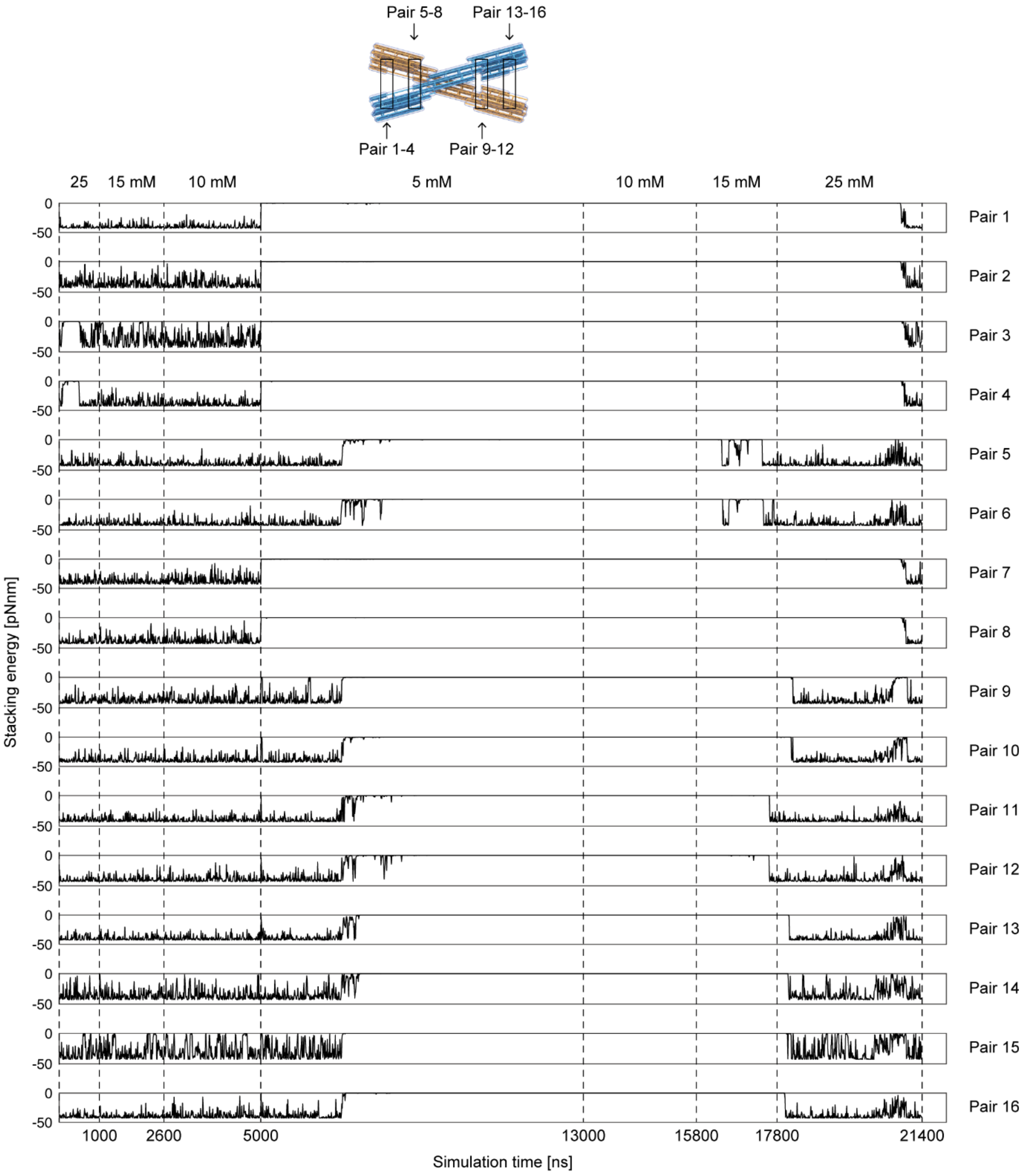

Supplementary Figure 15. Trajectory of stacking energy in the switch structure

For 16 stacking sites, the energy profile was computed respectively. The location of the stacking base-pairs is identical to that of the MD-simulated structures.

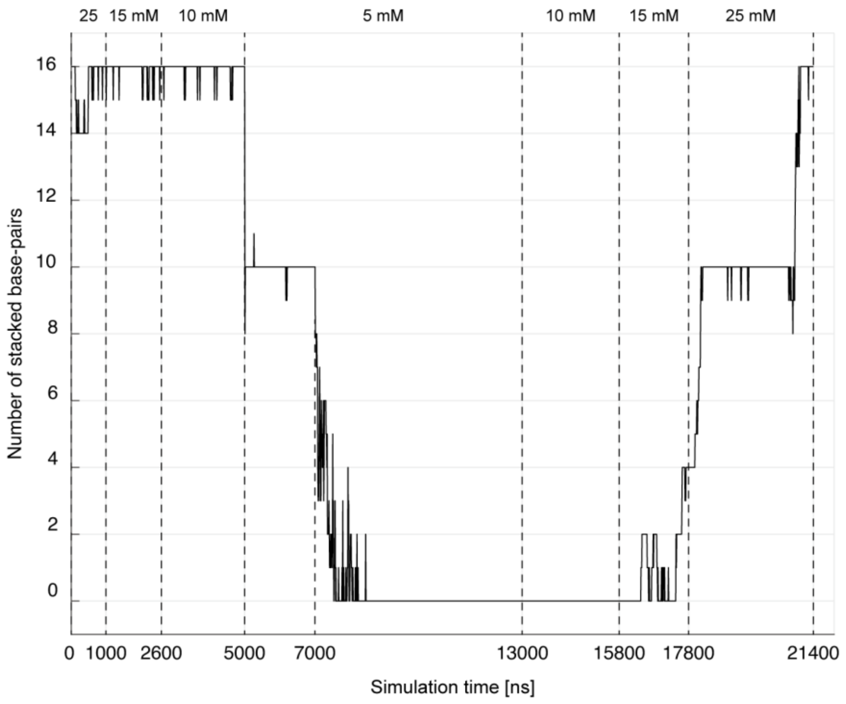

Supplementary Figure 16. The number of stacked base-pairs in the switch structure

Each stacking site was considered stacked (1) or unstacked (0) when its stacking energy was less or greater than -1 pNnm, respectively. The sum of the values for 16 stacking sites is illustrated.

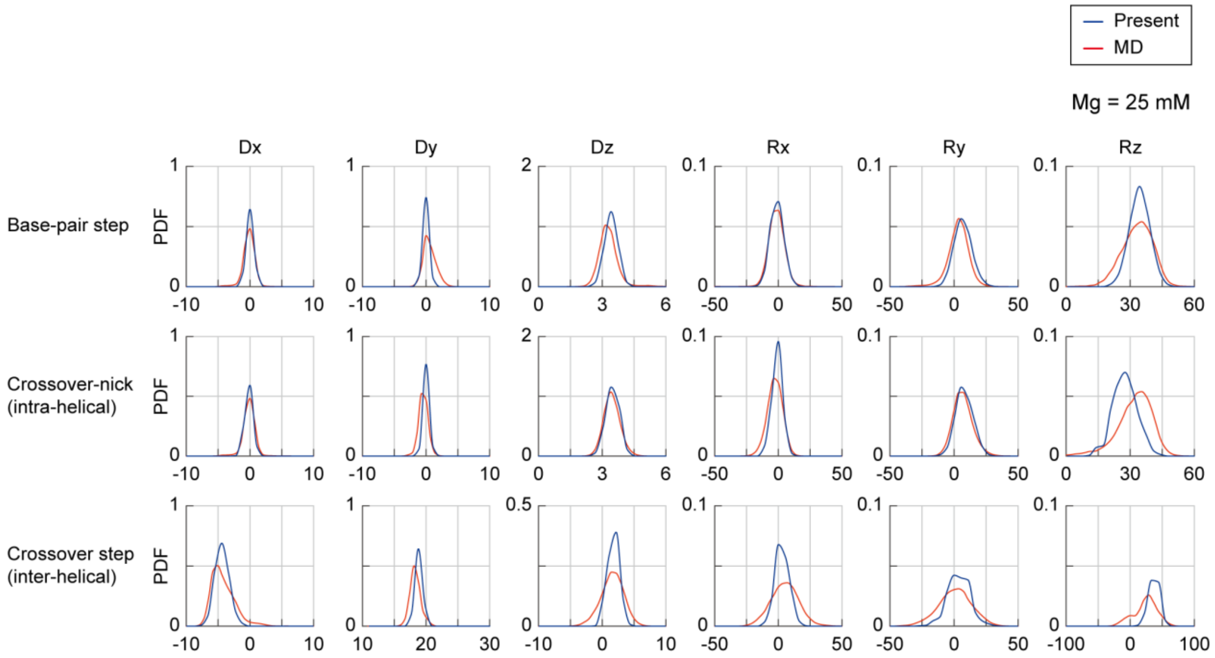

Supplementary Figure 17. Local geometry of the switch structure at Mg^{2+} 25 mM

The geometric values of base-pair steps and crossovers were calculated using the trajectories from the proposed and MD simulations at the salt concentration of Mg^{2+} 25 mM, respectively.

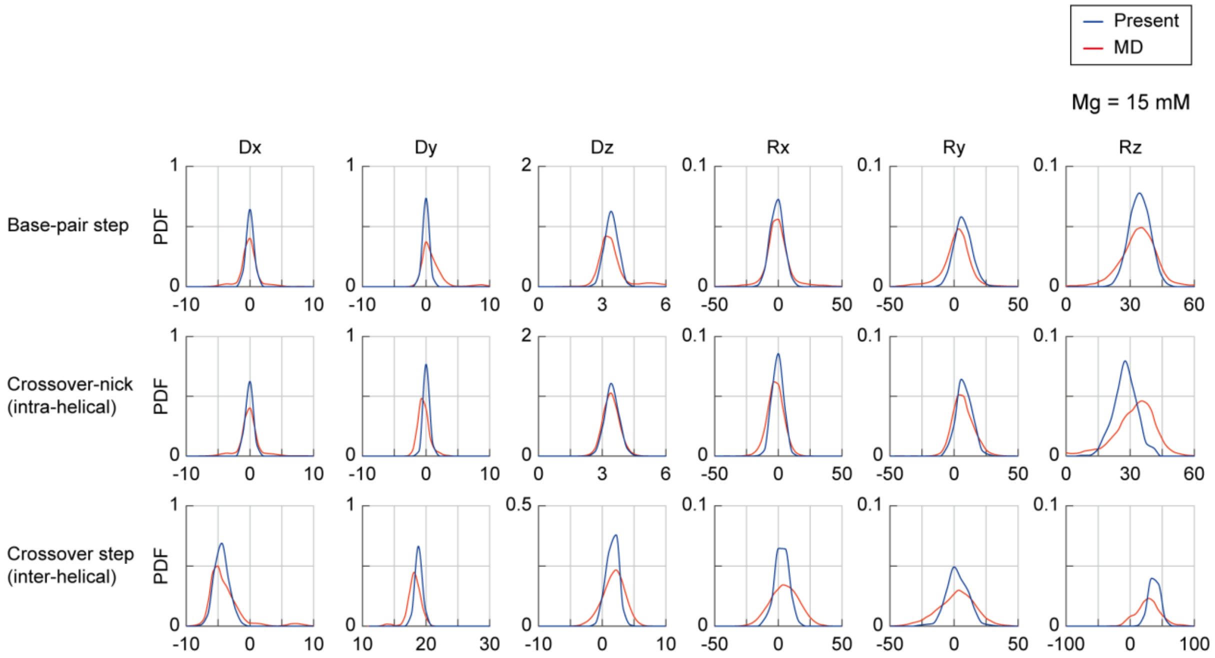

Supplementary Figure 18. Local geometry of the switch structure at Mg^{2+} 15 mM

The geometric values of base-pair steps and crossovers were calculated using the trajectories from the proposed and MD simulations at the salt concentration of Mg^{2+} 15 mM, respectively.

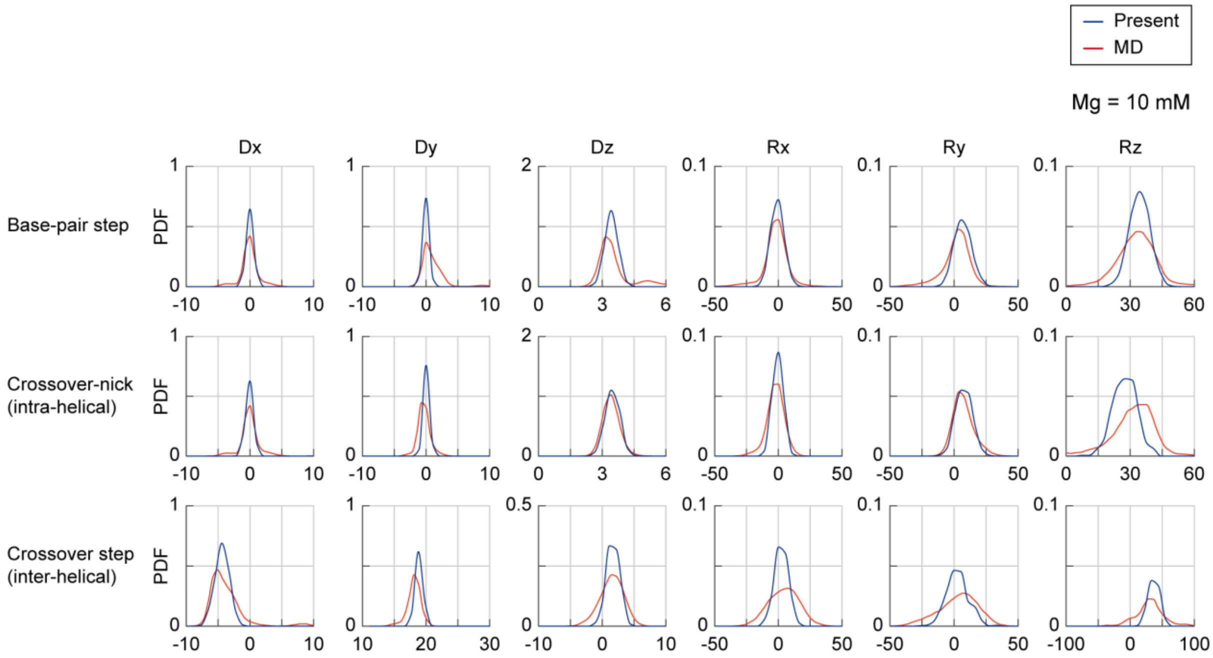

Supplementary Figure 19. Local geometry of the switch structure at Mg^{2+} 10 mM

The geometric values of base-pair steps and crossovers were calculated using the trajectories from the proposed and MD simulations at the salt concentration of Mg^{2+} 10 mM, respectively.

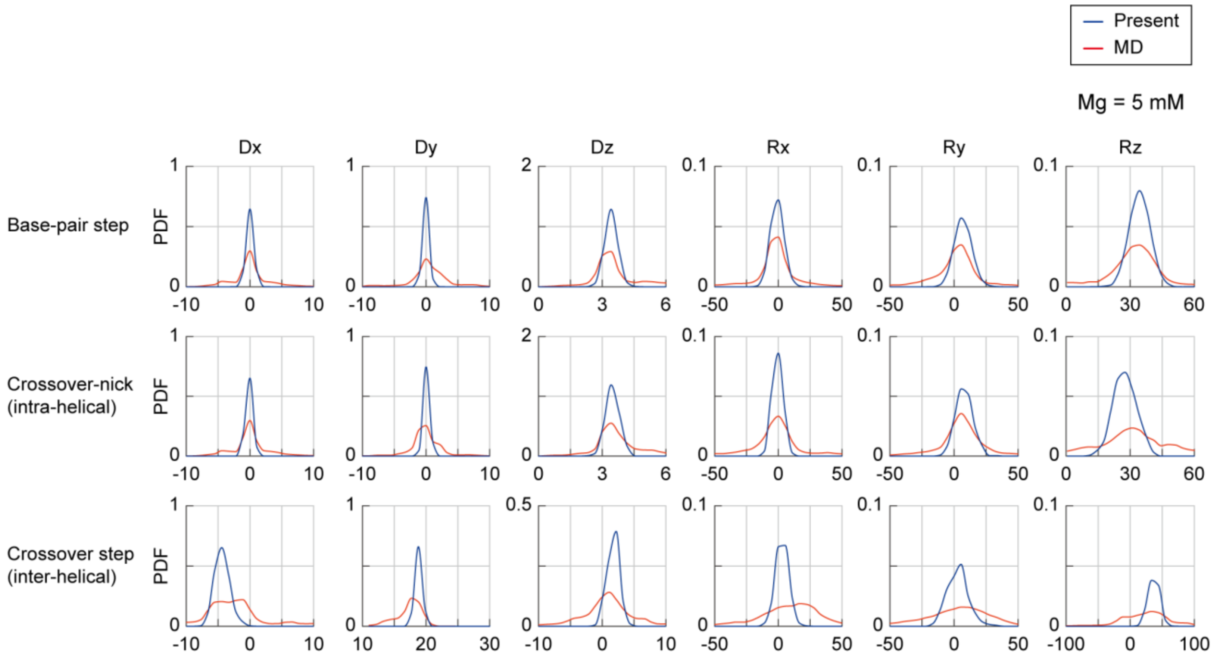

Supplementary Figure 20. Local geometry of the switch structure at Mg^{2+} 5 mM

The geometric values of base-pair steps and crossovers were calculated using the trajectories from the proposed and MD simulations at the salt concentration of Mg^{2+} 5 mM, respectively.

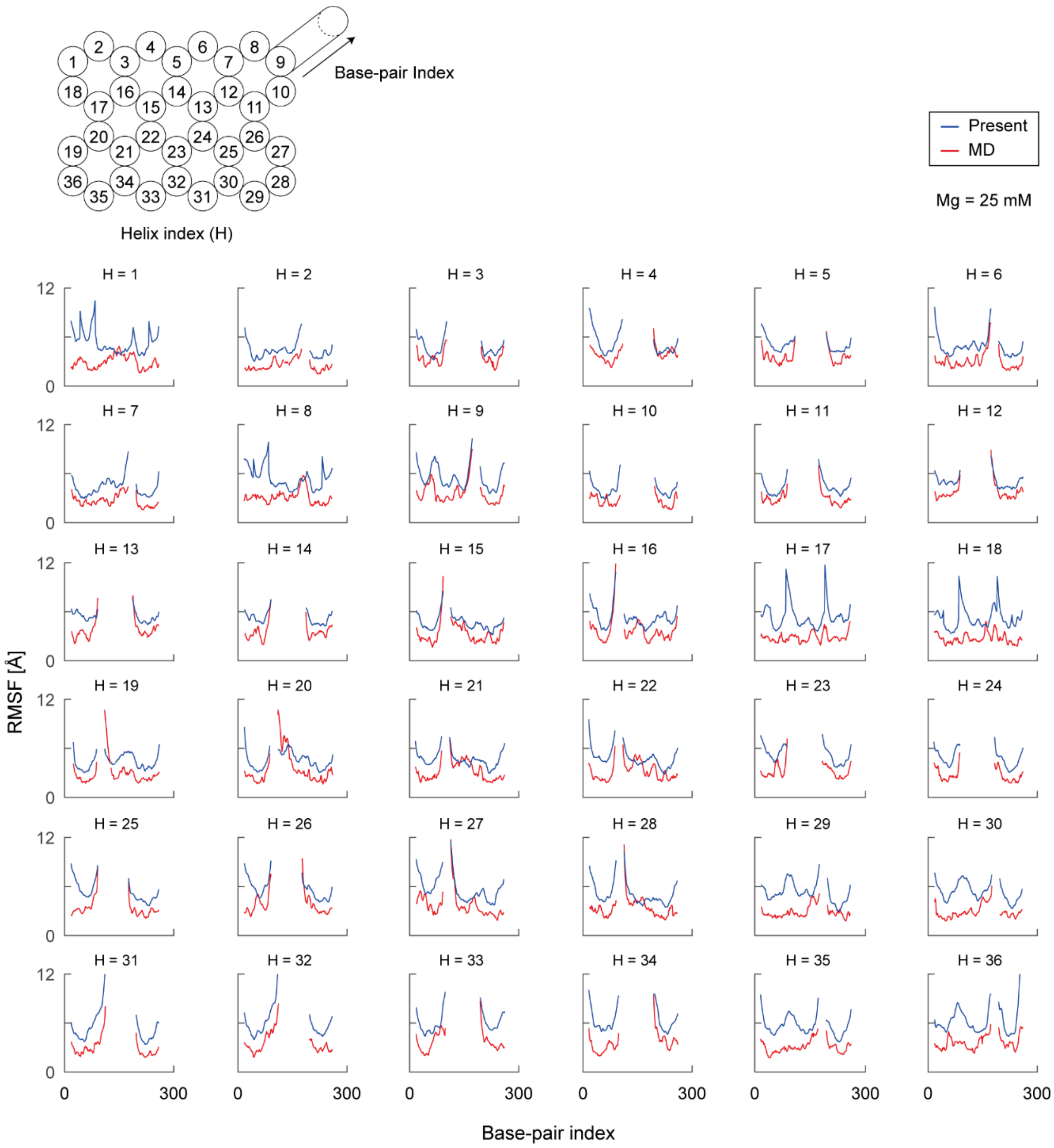

Supplementary Figure 21. RMSF distribution of the switch structure at Mg^{2+} 25 mM

The positional trajectory of each node (base-pair) was collected and converted to the RMSF from the proposed and MD simulations at the salt concentration of Mg^{2+} 25 mM, respectively. Before calculating RMSF values, the frames of the switch structure were aligned to the initial configuration.

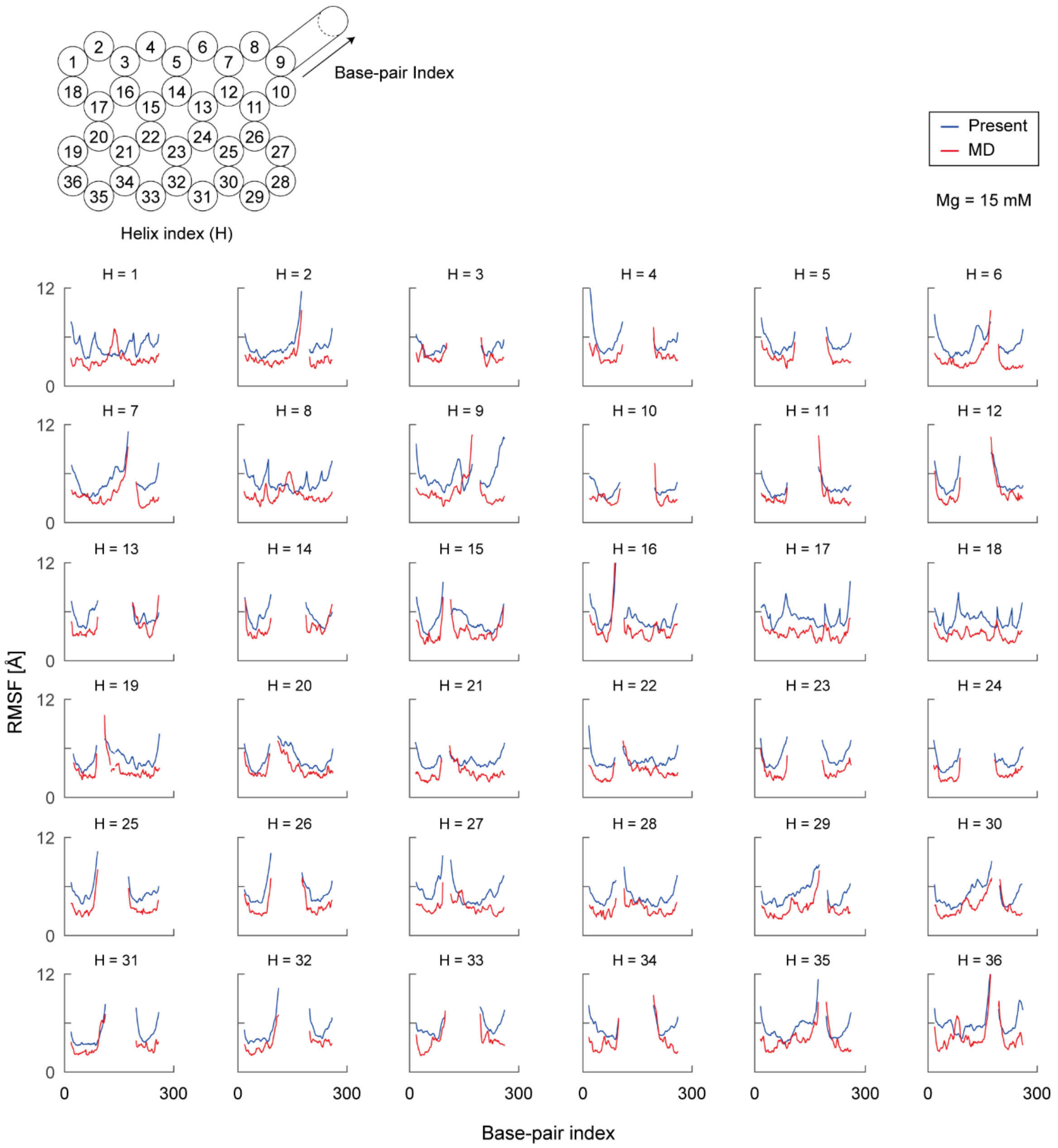

Supplementary Figure 22. RMSF distribution of the switch structure at Mg^{2+} 15 mM

The positional trajectory of each node (base-pair) was collected and converted to the RMSF from the proposed and MD simulations at the salt concentration of Mg^{2+} 15 mM, respectively. Before calculating RMSF values, the frames of the switch structure were aligned to the initial configuration.

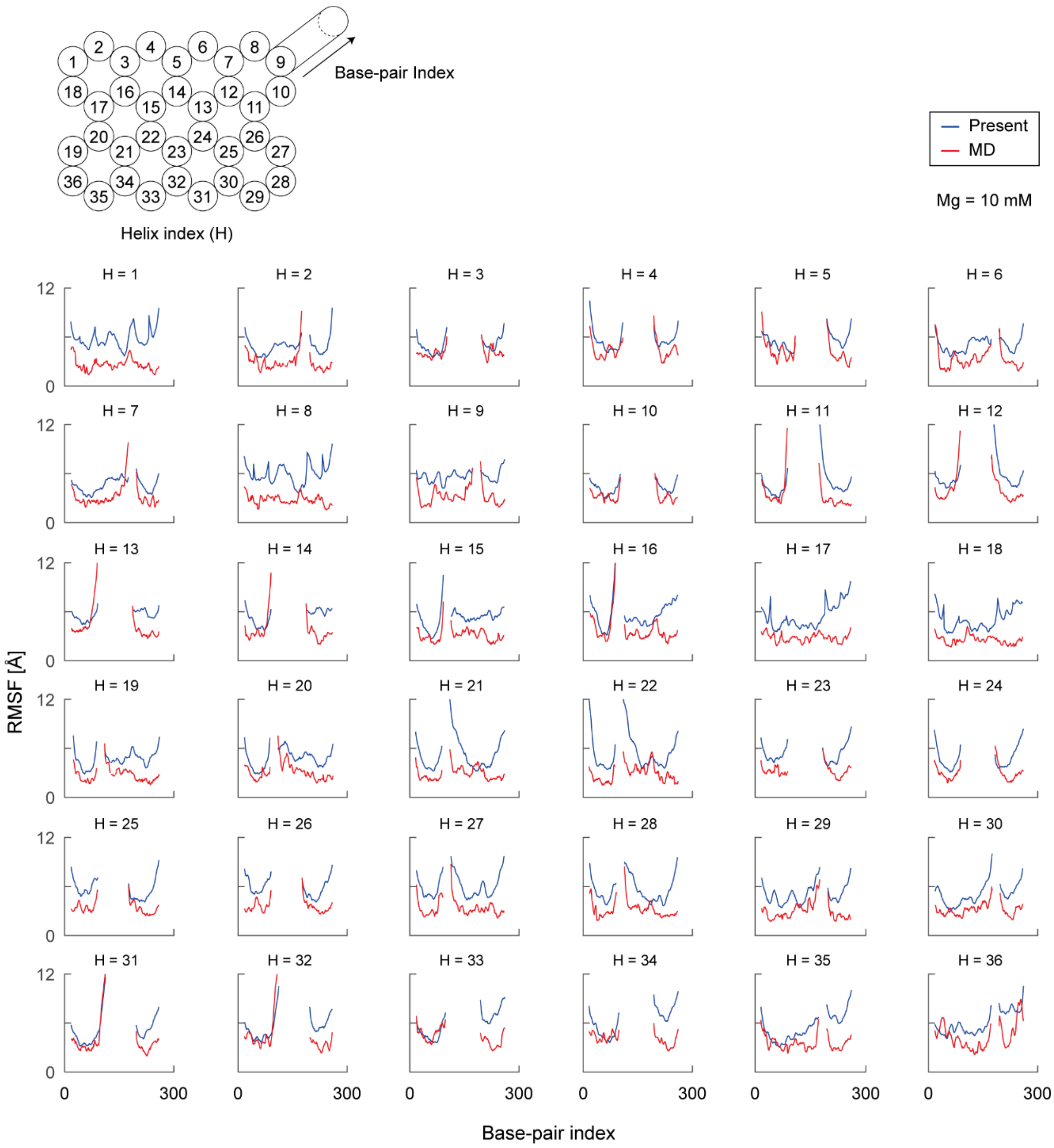

Supplementary Figure 23. RMSF distribution of the switch structure at Mg^{2+} 10 mM

The positional trajectory of each node (base-pair) was collected and converted to the RMSF from the proposed and MD simulations at the salt concentration of Mg^{2+} 10 mM, respectively. Before calculating RMSF values, the frames of the switch structure were aligned to the initial configuration.

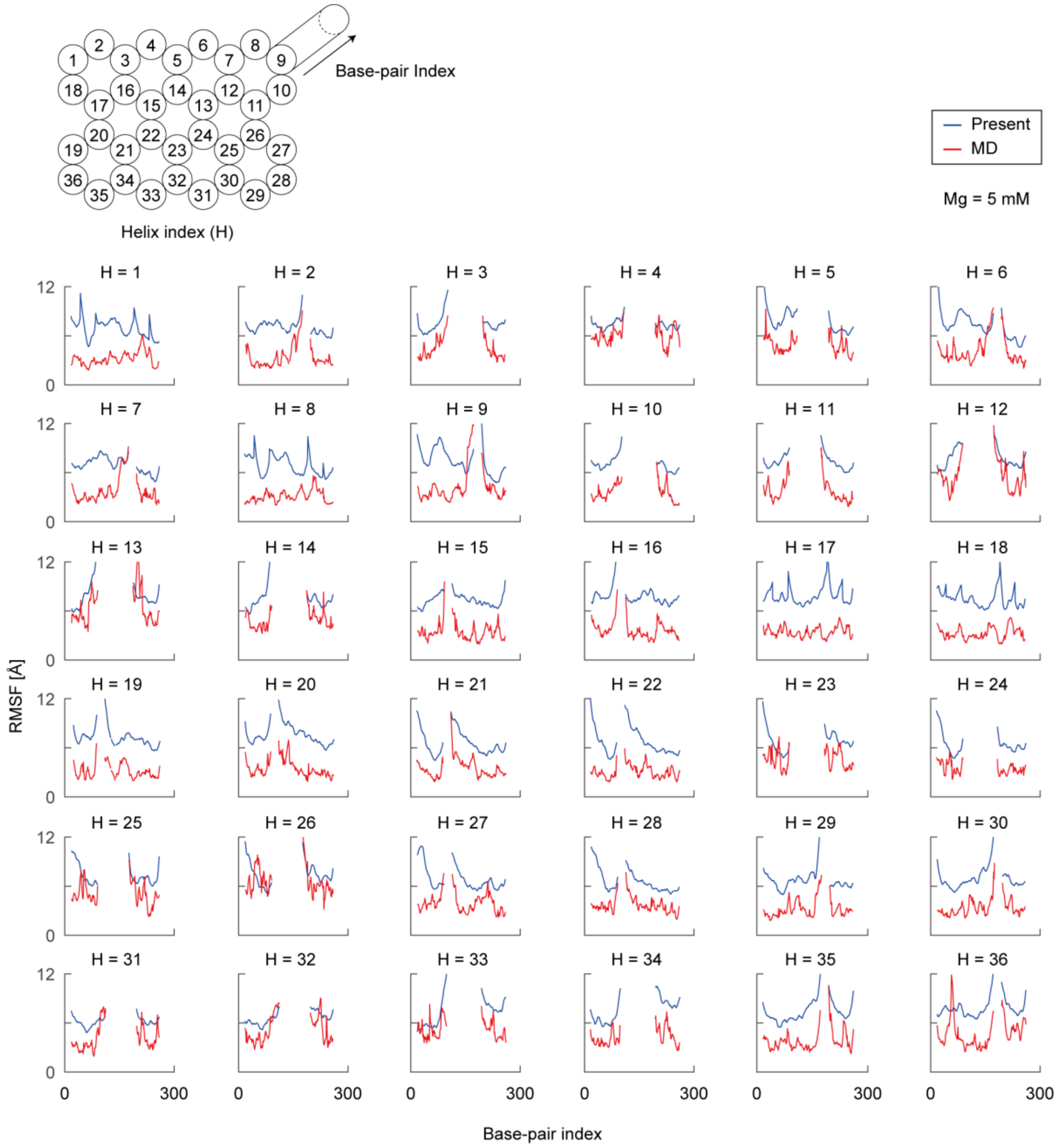

Supplementary Figure 24. RMSF distribution of the switch structure at Mg^{2+} 5 mM

The positional trajectory of each node (base-pair) was collected and converted to the RMSF from the proposed and MD simulations at the salt concentration of Mg^{2+} 5 mM, respectively. Before calculating RMSF values, the frames of the switch structure were aligned to the initial configuration.

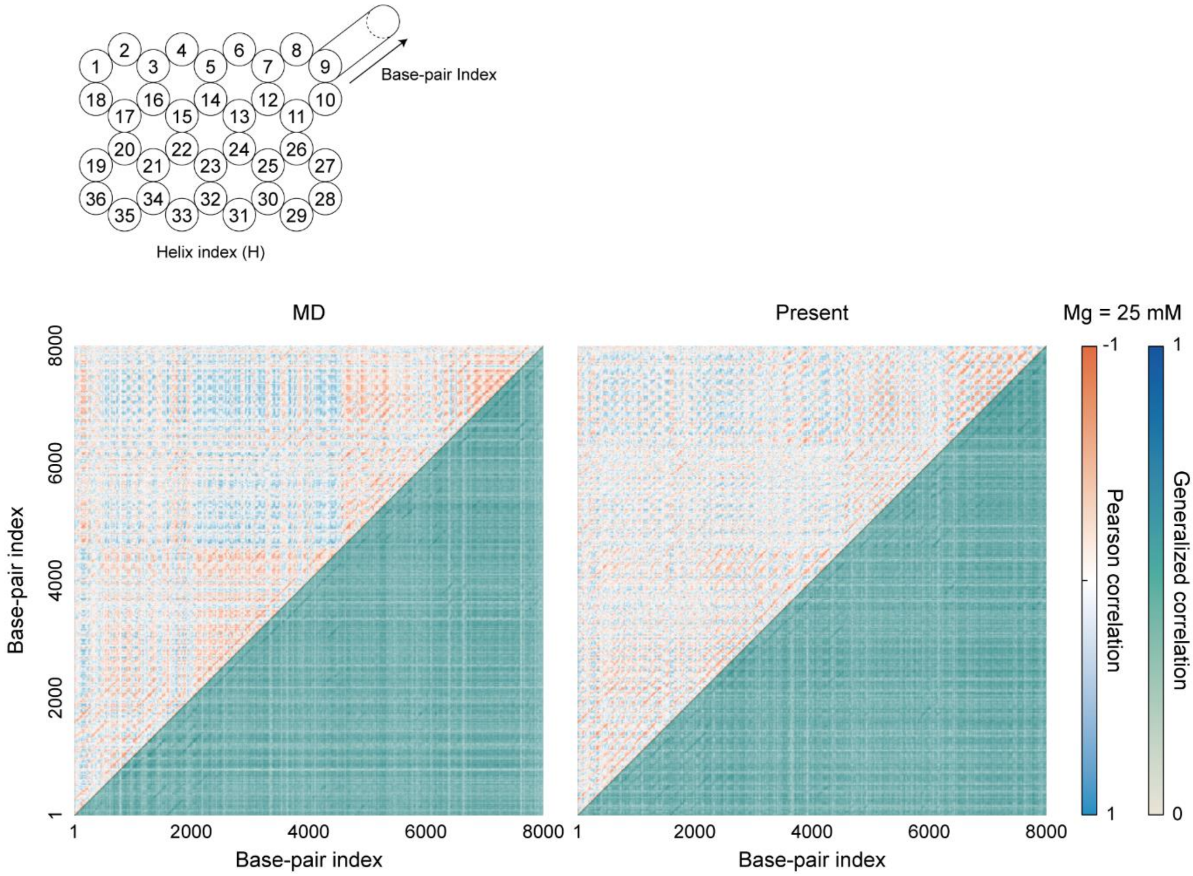

Supplementary Figure 25. Correlation maps of the switch structure at Mg^{2+} 25 mM

The positional trajectory of each node (base-pair) generated the Pearson and generalized correlation maps. We used the trajectories from the proposed and MD simulations at the salt concentration of Mg^{2+} 25 mM, respectively.

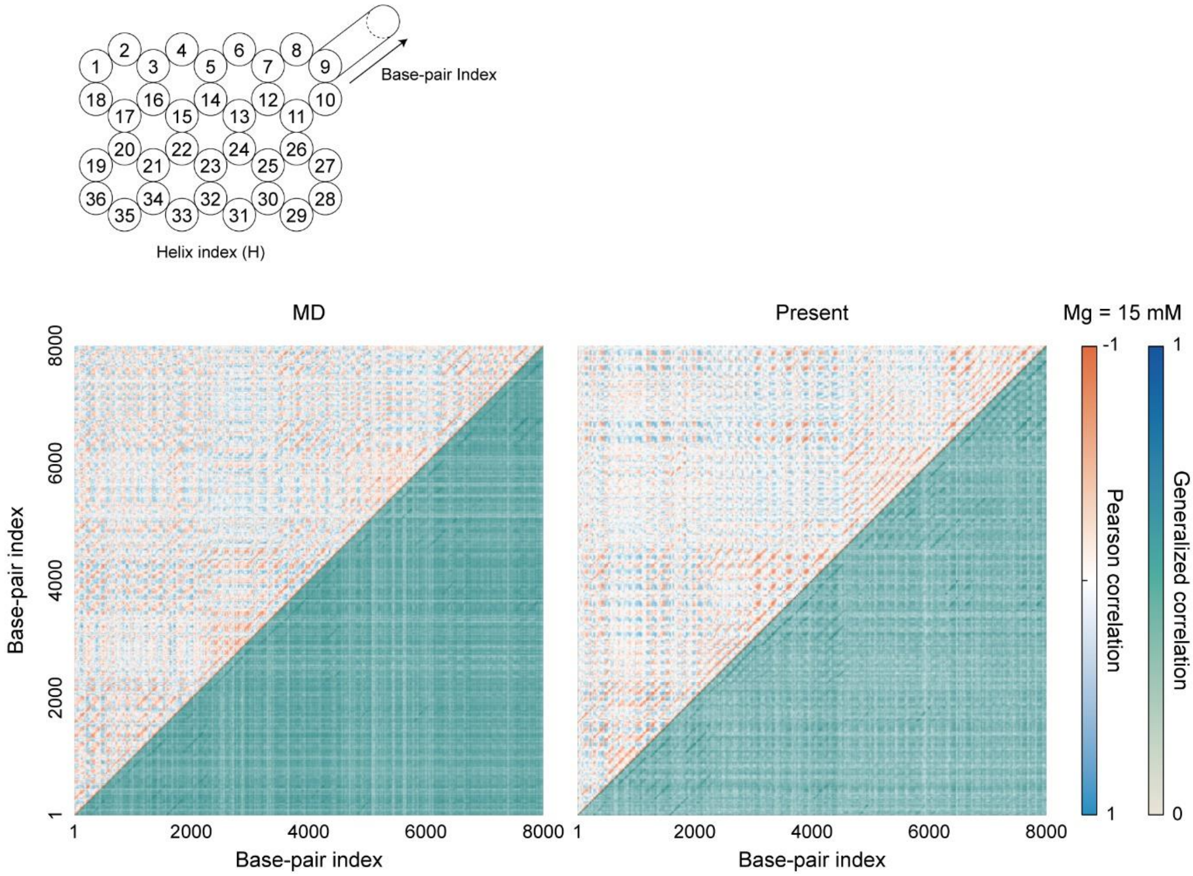

Supplementary Figure 26. Correlation maps of the switch structure at Mg^{2+} 15 mM

The positional trajectory of each node (base-pair) generated the Pearson and generalized correlation maps. We used the trajectories from the proposed and MD simulations at the salt concentration of Mg^{2+} 15 mM, respectively.

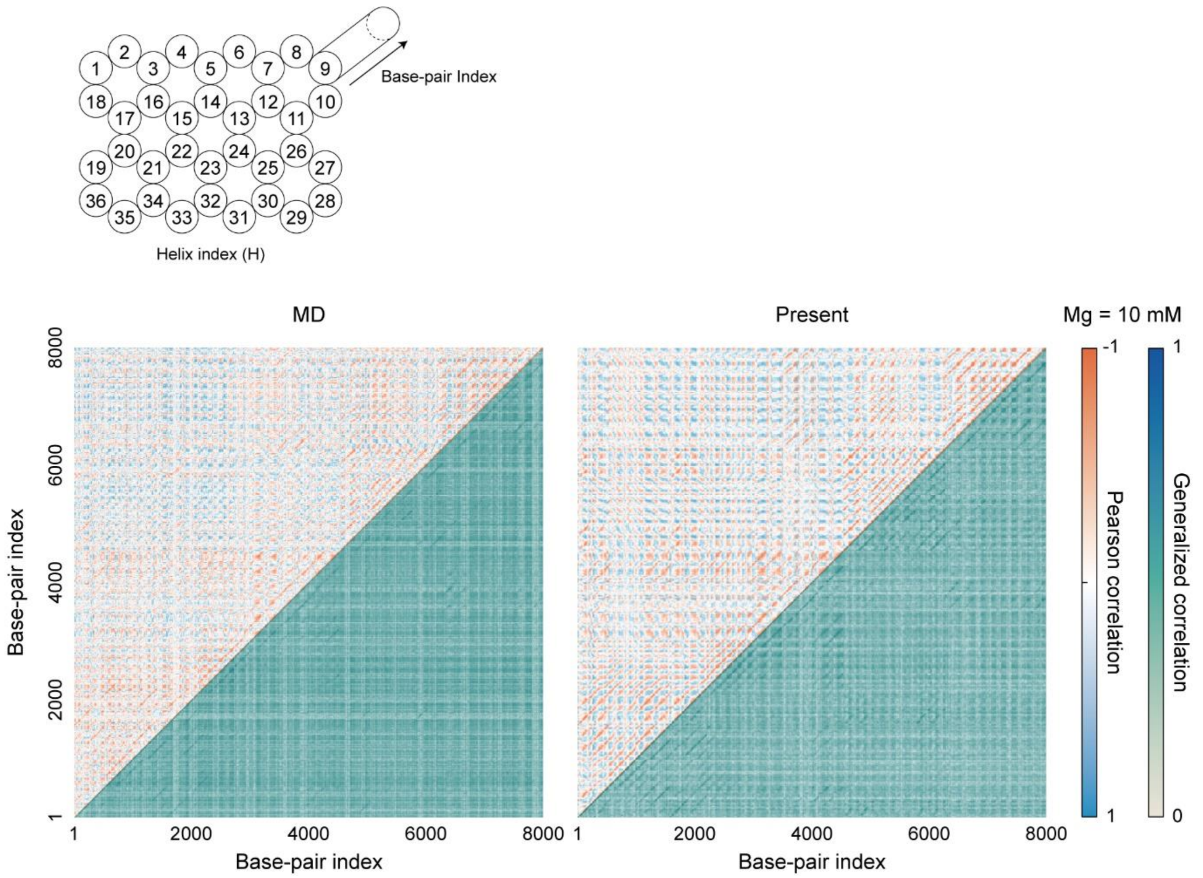

Supplementary Figure 27. Correlation maps of the switch structure at Mg^{2+} 10 mM

The positional trajectory of each node (base-pair) generated the Pearson and generalized correlation maps. We used the trajectories from the proposed and MD simulations at the salt concentration of Mg^{2+} 10 mM, respectively.

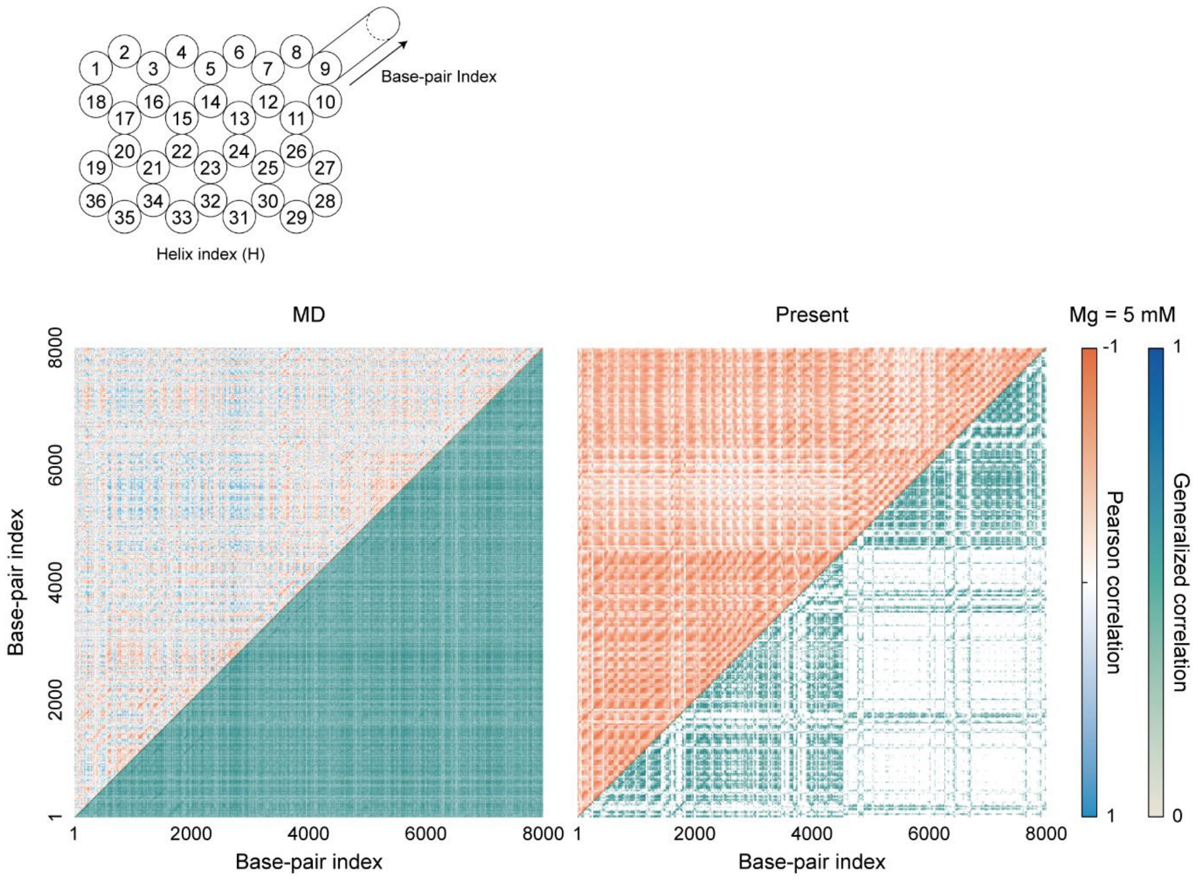

Supplementary Figure 28. Correlation maps of the switch structure at Mg^{2+} 5 mM

The positional trajectory of each node (base-pair) generated the Pearson and generalized correlation maps. We used the trajectories from the proposed and MD simulations at the salt concentration of Mg^{2+} 5 mM, respectively.

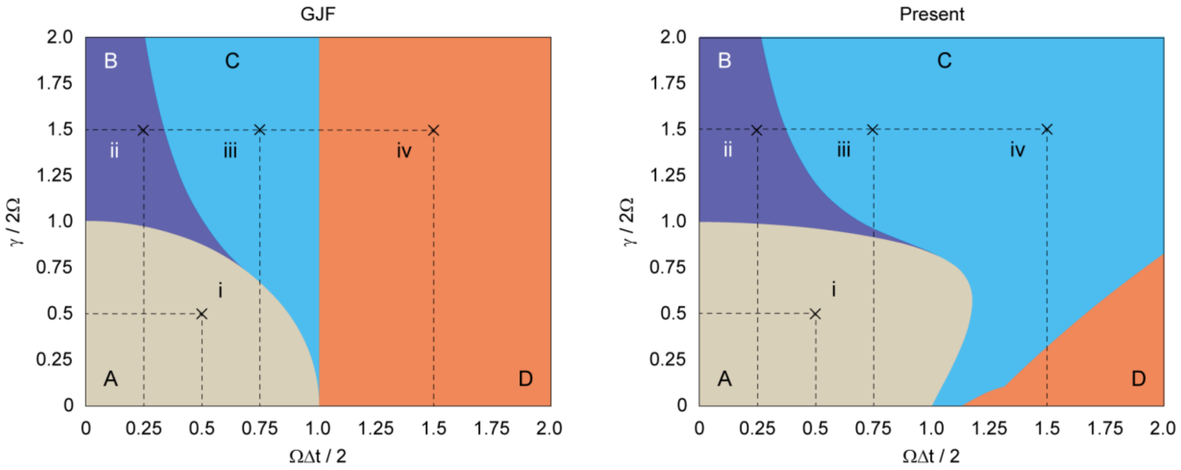

Supplementary Figure 29. Stability region of a thermal harmonic oscillator.

The stability region of the two algorithms was shown for two parameters (ΩΔt/2 and γ/2Ω) of a thermal harmonic oscillator. The characteristic regions were demonstrated as four regions given by

(A) $\Lambda_{\pm}$ are complex (Underdamping)

(B) $\Lambda_{\pm}$ are real and $|\Lambda_{\pm}| < 1$ (Overdamping)

(C) $\Lambda_{\pm}$ are real and $\Lambda_{+}\Lambda_{-} < 1$

(D) $\Lambda_{\pm}$ are real and $|\Lambda_{\pm}| > 1$ (Numerically unstable)

The algorithms were numerically tested for the four different conditions given by

(i) $[\mathrm{m},\ \mathrm{k},\ \gamma,\ \Delta t,\ \Omega\Delta t/2,\ \gamma/2\Omega] = [1.0, 1.0, 1.0, 1.0, 0.50, 0.50]$

(ii) $[\mathrm{m},\ \mathrm{k},\ \gamma,\ \Delta t,\ \Omega\Delta t/2,\ \gamma/2\Omega] = [1.0, 1.0, 3.0, 0.5, 0.25, 1.50]$

(iii) $[\mathrm{m},\ \mathrm{k},\ \gamma,\ \Delta t,\ \Omega\Delta t/2,\ \gamma/2\Omega] = [1.0, 1.0, 3.0, 1.5, 0.75, 1.50]$

(iv) $[\mathrm{m},\ \mathrm{k},\ \gamma,\ \Delta t,\ \Omega\Delta t/2,\ \gamma/2\Omega] = [1.0, 1.0, 3.0, 3.0, 1.50, 1.50]$

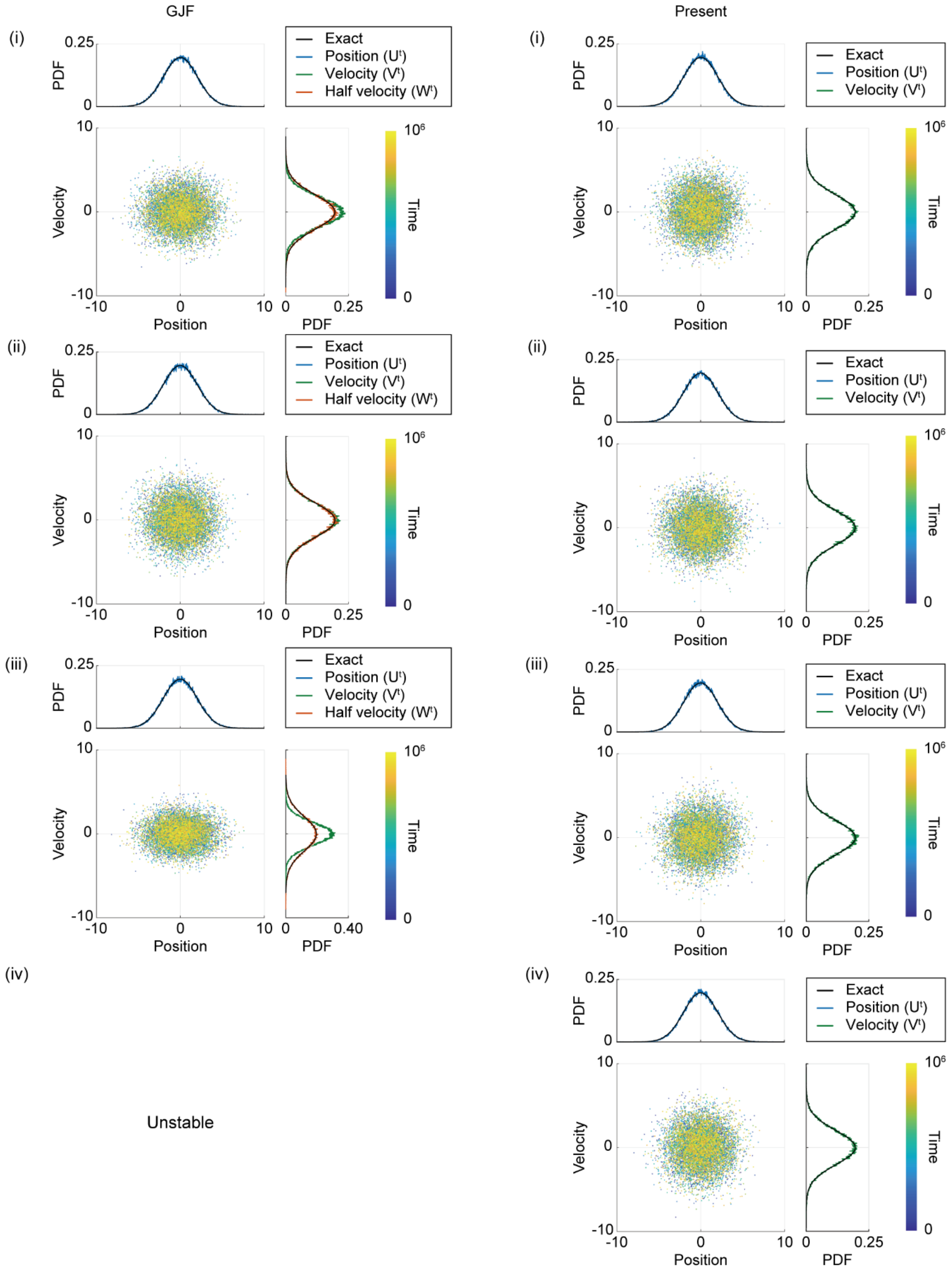

Supplementary Figure 30. Numerical tests of a thermal harmonic oscillator

The configuration and velocity distributions were calculated for the i, ii, iii, and iv conditions in Supplementary Figure 29.

Supplementary Tables

Supplementary Table 1. RMSD of wireframe structures to static prediction for final 100 ns

Structure		RMSD [Å] (mean & std)	
DX edge	Triangle (42-bp)	10.2	1.9
	Triangle (84-bp)	15.5	1.9
	Triangle (128-bp)	28.8	6.3
	Square (105-bp)	38.9	4.6
	Hexagon (74-bp)	44.5	2.1
6HB edge	Triangle (42-bp)	7.4	0.9
	Triangle (84-bp)	8.0	1.1
	Triangle (128-bp)	9.8	0.9
	Square (105-bp)	18.2	4.1
	Hexagon (74-bp)	14.3	1.9

Supplementary Table 2. Interior angle of wireframe structures

Structure		Present [°] (mean & std)		Exp[22] [°] (mean & std)	
DX edge	Triangle (128-bp)	59.1	6.1	63.4	13.9
	Square (105-bp)	99.3	10.5	-	-
	Hexagon (74-bp)	123.4	14.3	119.9	15.3
6HB edge	Triangle (128-bp)	62.5	3.0	61.3	4.0
	Square (105-bp)	98.9	8.3	90.0	8.1
	Hexagon (74-bp)	126.7	5.7	120.1	6.0

Supplementary Table 3. Out-of-plane angle of wireframe structures

Structure		Present [°] (mean & std)		MD[22] [°] (mean & std)	
DX edge	Triangle (42-bp)	12.6	3.4	15.5	3.7
	Triangle (84-bp)	11.6	2.7	7.2	1.7
6HB edge	Triangle (42-bp)	6.8	2.2	6.4	1.4
	Triangle (84-bp)	5.3	0.8	5.0	1.6

Supplementary Table 4. Natural frequency of the pointer structure

Mode number	Normal mode analysis [GHz]	Principal component analysis [GHz]
1	5.64E-07	0.22
2	2.55E-06	0.55
3	5.56E-06	1.07
4	1.83E-03	1.16
5	2.36E-03	1.85
6	2.51E-03	2.35
7	3.22	4.53
8	4.59	4.92
9	4.71	5.22
10	5.24	5.56
11	5.49	5.65
12	6.00	5.88
13	6.54	6.12
14	6.95	6.26
15	7.26	6.36
16	7.72	6.56
17	7.90	6.72
18	8.30	6.94
19	8.50	7.04
20	9.06	7.05
21	9.45	7.10
22	9.68	7.23
23	9.79	7.29
24	10.13	7.40
25	10.28	7.50
26	10.52	7.52
27	10.87	7.65
28	11.22	7.70
29	11.24	7.81
30	11.50	7.86

Supplementary Table 5. System of MD simulations for the switch structure

Simulation box [nm^3]	Water number	Ion concentration [mM]			Ion number		
		Mg^{2+}	Na^{+}	Cl^{-}	Mg^{2+}	Na^{+}	Cl^{-}
100 × 22 × 26	4,476,495	25	5	55	8,717	151	1,666
95 × 23 × 27	4,697,439	15	5	35	8,427	156	1,092
94 × 23 × 45	8,466,582	10	5	25	8,506	273	1,365
96 × 26 × 50	11,434,815	5	5	15	8,294	334	1,002

Supplementary Table 6. Computing environment and performance for switch structure

	Server	CPU	CPU cores	GPU	CUDA
**Present**	Dell T640<br>(physical 1 server)	Intel Xeon Silver 4210R 2.40GHz 2EA	20	NVIDIA RTX A6000 48GB (1EA)	11.6
**MD**	KISTI NURION<br>(CPU cluster)	Intel Xeon Phi 7250	24,000	-	-
	Dell C4130<br>(physical 1 server)	Intel Xeon Processor E5-2680 2.50 GHz 2EA	12	NVIDIA Tesla K80 24GB (4EA)	11.5

	Server	Mg^{2+} concentration [mM]	Simulation time [ns]	Wall time [h]	Sampling speed [ns/h]
**Present**	Dell T640	25	1000	26.9	37.2
		15	1600	45.1	35.5
		10	2400	65.8	36.5
		5	8000	226.0	35.4
		10	2800	77.9	35.9
		15	2000	55.5	36.0
		25	3600	97.9	36.8
**MD**	KISTI NURION	25	81.5	342.8	0.238
		15	77.4	327.7	0.236
		10	69.3	336.9	0.206
		5	18.6	116.8	0.159
	Dell C4130	5	20.0	1601.8	0.012

Supplementary References

1 Tuckerman, M. *Statistical mechanics: theory and molecular simulation*. (Oxford Univ. Press, Oxford, 2010).

2 Lee, J. Y. *et al.* Rapid computational analysis of DNA origami assemblies at near-atomic resolution. *ACS Nano* **15**, 1002-1015 (2021).

3 Lee, J. Y. *et al.* Investigating the sequence-dependent mechanical properties of DNA nicks for applications in twisted DNA nanostructure design. *Nucleic Acids Res.* **47**, 93-102 (2019).

4 Lee, J. Y., Kim, M., Lee, C. & Kim, D. N. Characterizing and harnessing the mechanical properties of short single-stranded DNA in structured assemblies. *ACS Nano* **15**, 20430-20441 (2021).

5 Battini, J. M. & Pacoste, C. Co-rotational beam elements with warping effects in instability problems. *Comput. Methods. Appl. Mech. Eng.* **191**, 1755-1789 (2002).

6 Muddiman, D. C., Anderson, G. A., Hofstadler, S. A. & Smith, R. D. Length and base composition of PCR-amplified nucleic acids using mass measurements from electrospray ionization mass spectrometry. *Anal. Chem.* **69**, 1543-1549 (1997).

7 Wajnryb, E., Mizerski, K. A., Zuk, P. J. & Szymczak, P. Generalization of the Rotne–Prager–Yamakawa mobility and shear disturbance tensors. *J. Fluid Mech.* **731** (2013).

8 Rotne, J. & Prager, S. Variational treatment of hydrodynamic interaction in polymers. *J. Chem. Phys.* **50**, 4831-& (1969).

9 Yamakawa, H. Transport properties of polymer chains in dilute solution: hydrodynamic interaction. *J. Chem. Phys.* **53**, 436-& (1970).

10 Sedeh, R. S. *et al.* Computing nonequilibrium conformational dynamics of structured nucleic acid assemblies. *J. Chem. Theory. Comput.* **12**, 261-273 (2016).

11 Kestin, J., Sokolov, M. & Wakeham, W. A. Viscosity of liquid water in the range −8°C to 150°C. *J. Phys. Chem. Ref. Data* **7**, 941-948 (1978).

12 Durlofsky, L., Brady, J. F. & Bossis, G. Dynamic simulation of hydrodynamically interacting particles. *J. Fluid Mech.* **180**, 21-49 (1987).

13 Grønbech-Jensen, N. & Farago, O. A simple and effective Verlet-type algorithm for simulating Langevin dynamics. *Mol. Phys.* **111**, 983-991 (2013).

14 Ermak, D. L. & Mccammon, J. A. Brownian dynamics with hydrodynamic interactions. *J. Chem. Phys.* **69**, 1352-1360 (1978).

15 Cichocki, B., Jones, R. B., Kutteh, R. & Wajnryb, E. Friction and mobility for colloidal spheres in Stokes flow near a boundary: The multipole method and applications. *J. Chem. Phys.* **112**, 2548-2561 (2000).

16 Farago, O. Langevin thermostat for robust configurational and kinetic sampling. *Phys. A: Stat. Mech. Appl.* **534**, 122210 (2019).

17 Leimkuhler, B. & Matthews, C. Efficient molecular dynamics using geodesic integration and solvent-solute splitting. *Proc. Math. Phys. Eng. Sci.* **472**, 20160138 (2016).

18 Kube, M. *et al.* Revealing the structures of megadalton-scale DNA complexes with nucleotide resolution. *Nat. Commun.* **11**, 6229 (2020).

19 Brooks, B. R., Janežič, D. & Karplus, M. Harmonic analysis of large systems. I. Methodology. *J. Comput. Chem.* **16**, 1522-1542 (1995).

20 Snodin, B. E. *et al.* Introducing improved structural properties and salt dependence into a coarse-grained model of DNA. *J. Chem. Phys.* **142**, 234901 (2015).

21 Yagyu, H., Lee, J. Y., Kim, D. N. & Tabata, O. Coarse-grained molecular dynamics model of double-stranded DNA for DNA nanostructure design. *J. Phys. Chem. B* **121**, 5033-5039 (2017).

22 Jun, H. *et al.* Autonomously designed free-form 2D DNA origami. *Sci. Adv.* **5**, eaav0655 (2019).

23 Lee, J. G., Kim, K. S., Lee, J. Y. & Kim, D. N. Predicting the free-form shape of structured DNA assemblies from their lattice-based design blueprint. *ACS Nano* **16**, 4289-4297 (2022).

24 Jun, H., Wang, X., Bricker, W. P. & Bathe, M. Automated sequence design of 2D wireframe DNA origami with honeycomb edges. *Nat. Commun.* **10**, 5419 (2019).

25 Veneziano, R. *et al.* Designer nanoscale DNA assemblies programmed from the top down. *Science* **352**, 1534 (2016).

26 Humphrey, W., Dalke, A. & Schulten, K. VMD: visual molecular dynamics. *J. Mol. Graphics* **14**, 33-38 (1996).
27 Wang, X. *et al.* Planar 2D wireframe DNA origami. *Sci. Adv.* **8**, eabn0039 (2022).
28 Wang, D. *et al.* Programmable transformations of DNA Origami made of small modular dynamic units. *J. Am. Chem. Soc.* **143**, 2256-2263 (2021).
29 Gur, F. N. *et al.* Double- to single-strand transition induces forces and motion in DNA origami nanostructures. *Adv. Mater.* **33**, e2101986 (2021).
30 Bai, X. C., Martin, T. G., Scheres, S. H. & Dietz, H. Cryo-EM structure of a 3D DNA-origami object. *Proc. Natl. Acad. Sci. U. S. A.* **109**, 20012-20017 (2012).
31 Lee, C., Lee, J. Y. & Kim, D. N. Polymorphic design of DNA origami structures through mechanical control of modular components. *Nat. Commun.* **8**, 2067 (2017).
